# Low-intensity pulsed ultrasound (LIPUS) enhances the anti-inflammatory effects of bone marrow mesenchymal stem cells (BMSCs)-derived extracellular vesicles

**DOI:** 10.1186/s11658-023-00422-3

**Published:** 2023-01-30

**Authors:** Xueke Li, Yi Zhong, Wuqi Zhou, Yishu Song, Wenqu Li, Qiaofeng Jin, Tang Gao, Li Zhang, Mingxing Xie

**Affiliations:** 1 Department of Ultrasound Medicine, Union Hospital, Tongji Medical College, Huazhong University of Science and Technology, Wuhan, 430022 China; 2 Clinical Research Center for Medical Imaging in Hubei Province, Wuhan, 430022 China; 3 Hubei Province Key Laboratory of Molecular Imaging, Wuhan, 430022 China

**Keywords:** Low-intensity pulsed ultrasound, Extracellular vesicles, Inflammation, Bone marrow mesenchymal stem cells, microRNA

## Abstract

**Background:**

Bone marrow-derived mesenchymal stem cells (BMSCs)-derived extracellular vesicles (EVs) have shown potent anti-inflammatory function in various pathological conditions, such as osteoarthritis and neurodegenerative diseases. Since the number of EVs naturally secreted by cells is finite and they usually bear specific repertoires of bioactive molecules to perform manifold cell–cell communication, but not one particular therapeutic function as expected, their practical application is still limited. Strategies are needed to increase the production of EVs and enhance their therapeutic function. Recent studies have suggested that low-intensity pulsed ultrasound (LIPUS) is a promising non-invasive method to increase the secretion of EVs and promote their anti-inflammatory effects. However, the effect of LIPUS stimulation of BMSCs on EVs derived from the cells remains unclear. The objective of this study was to investigate whether LIPUS stimulation on BMSCs could increase the secretion of EVs and enhance their anti-inflammatory effects.

**Methods:**

BMSCs were exposed to LIPUS (300 mW/cm^2^) for 15 min and EVs were isolated by ultracentrifugation. Anti-inflammatory effects of EVs were investigated on RAW264.7 cells in vitro and in the allogeneic skin transplantation model. Small RNA-seq was utilized to identify components difference in EVs with/without LIPUS irradiation.

**Results:**

In this study, we found that LIPUS stimulation could lead to a 3.66-fold increase in the EVs release from BMSCs. Moreover, both in vitro and in vivo experimental results suggested that EVs secreted from LIPUS-treated BMSCs (LIPUS-EVs) possessed stronger anti-inflammatory function than EVs secreted from BMSCs without LIPUS stimulation (C-EVs). RNA-seq analysis revealed that miR-328-5p and miR-487b-3p were significantly up-regulated in LIPUS-EVs compare with C-EVs. The suppression of MAPK signaling pathway by these two up-regulated miRNAs could be the potential mechanism of strengthened anti-inflammatory effects of LIPUS-EVs.

**Conclusion:**

LIPUS stimulation on BMSCs could significantly increase the secretion of EVs. Moreover, EVs generated from LIPUS-treated BMSCs possessed much stronger anti-inflammatory function than C-EVs. Therefore, LIPUS could be a promising non-invasive strategy to promote the production of EVs from BMSCs and augment their anti-inflammatory effects.

**Supplementary Information:**

The online version contains supplementary material available at 10.1186/s11658-023-00422-3.

## Background

Mesenchymal stem cells (MSCs)-derived extracellular vesicles (EVs) have been reported to show potent anti-inflammatory effects in a variety of diseases such as osteoarthritis, cardiovascular diseases, graft-versus-host disease (GVHD), and neurodegenerative diseases [1–5]. As nanoscale non-reproducible lipid bilayer particles, EVs are naturally released by most, if not all, types of cells and are present in most bodily fluids [6, 7]. They inherit the therapeutic effects from their parent cells by containing sufficient bioactive molecules, including lipids, proteins and nucleic acids such as miRNAs in a phospholipid bilayer membrane, while evade the safety concerns associated with cell therapy [8–10]. This advantage makes them promising novel therapeutics for inflammation [6, 11], thus leading to recent surge of interest in EVs [12–14]. Nowadays, their biology is better understood and their potential in serving as diagnostic and therapeutic tools is more appreciated [15]. However, the practical application of EVs is still limited due to the relatively low yield and restricted therapeutic constituents [16, 17]. While consistent large-scale supply of EVs is essential for actual practice, especially for clinical application, the number of EVs secreted by the cells is finite [18, 19]. Comparison among a variety of available EVs isolation methods indicates that EVs separation methods with high purity always result in lower yield [20, 21]. Moreover, EVs naturally secreted by cells usually bear specific repertoires of bioactive molecules to perform manifold cell–cell communication, but not one particular therapeutic function as expected [22, 23]. Therefore, it is inevitable to seek an appropriate strategy to increase EVs secretion and even modify their components before EVs isolation. Ideally, combining such manipulation strategy with the gold standard isolation method (ultracentrifugation) will boost EVs yield and enhance their therapeutic efficacy without introducing harmful risks.

Low-intensity pulsed ultrasound (LIPUS), as its name suggests, is the administration of ultrasound at a considerably lower intensity (< 3 W/cm^2^) than regular ultrasound in the form of pulse wave. Its therapeutic benefits have attracted much attention over the past few decades [24]. Despite the lack of consistency in the values reported, the most widely used parameters for LIPUS are as follows: spatial average temporal average intensity of 30 mW/cm^2^, pulse repetition frequency of 1 kHz at 20% duty cycle, and pulse frequency of 1.5 MHz [25]. Recently, Yang et al. reported that LIPUS could promote the biogenesis and docking of EVs by upregulating associated genes [26]. In an in vitro study, Li et al*.* observed that EVs generated from LIPUS-treated bone marrow dendritic cells (BMDCs) possessed anti-inflammatory effects by containing higher amounts of miR-16 and miR-21 [27]. As a safe, convenient and low-cost physical method, LIPUS is a promising non-invasive strategy to regulate EVs production and promote their anti-inflammatory function.

The synergic function of LIPUS and MSCs in tissue regeneration has been investigated for decades [28, 29]. LIPUS can promote the differentiation of MSCs toward specific lineage once they are induced to a certain fate [28]. However, such multipotency of MSCs might not be their inherent property [30]. The studies of Dr. Bruno Peault and colleagues have clearly verified that MSCs are derived from pericytes [31]. Instead of differentiating into the tissue-specific cells at disease sites, MSCs migrate to the injured tissue and secrete bioactive factors once activated in vivo [32]. Therefore, MSCs are more like Medicinal Signaling Cells but not Mesenchymal Stem Cells. Such important medicinal role of MSCs needs further investigation. Since MSCs perform their immunomodulatory function mainly through paracrine, EVs as the major products of paracrine are pivotal for immunomodulation [3, 33]. But as we mentioned above, the low yield of EVs and restricted effective components for one specific therapeutic function hampers the practical application of MSCs-EVs. Fortunately, LIPUS is a promising strategy to solve such issue. Whether LIPUS stimulation on BMSCs could increase the secretion of EVs and enhance their anti-inflammatory effects is a significant question that needs to be answered.

Herein, we investigated the effects of LIPUS on the biogenesis and components of BMSCs-derived EVs and further analyzed whether LIPUS enhance the anti-inflammatory effects of BMSCs-derived EVs both on lipopolysaccharide (LPS)-induced macrophage and skin transplant mice. Moreover, the mechanism of LIPUS affecting BMSCs-derived EVs was explored using RNA-seq method. The results showed that LIPUS stimulation could promote the proliferation, EVs secretion and anti-inflammatory functions of BMSCs without affecting the morphology and apoptosis of the cells. And LIPUS stimulation could lead to a 3.66-fold increase in the EVs release from BMSCs. Interestingly, compared to the EVs derived from LIPUS-untreated BMSCs (the C-EVs), the EVs derived from LIPUS-treated BMSCs (the LIPUS-EVs) contained higher amount of miR-328-5p and miR-487b-3p, which could exert anti-inflammation functions by targeting genes in the MAPK signaling pathway. RAW264.7 cells in response to LPS and incubated with LIPUS-EVs expressed higher anti-inflammatory factor (IL-10) and had lower activity of nuclear factor (NF)-κB signaling than C-EVs group, suggesting that LIPUS stimulation strengthened the suppression of BMSCs-derived EVs on LPS-induced infectious inflammation. Moreover, the skin allografts in mice injected with LIPUS-EVs showed higher expression of IL-10, lower expression of pro-inflammatory factor (IL-6), and much fewer immune cells infiltration than C-EVs group, indicating that LIPUS irradiation strengthened the suppression of BMSCs-derived EVs on both infectious and sterile inflammation. Both in vitro and in vivo experiments confirmed that LIPUS could facilitate the anti-inflammatory effects of BMSCs-derived EVs. These results provide a theoretical foundation for applying LIPUS as a non-invasive strategy to increase the production of EVs from BMSCs as well as enhance their anti-inflammatory effect. Thus, the clinical translation of such cell-free therapy can be fostered.

## Methods

### Isolation and characterization of BMSCs

The BMSCs were harvested from 4-week-old C57 mice (male, 15–20 g). After intraperitoneal injection of pentobarbital sodium anesthesia, the femurs and tibiae of C57 mice were harvested immediately. Bone marrow was flushed from the bone cavity of the femurs and tibias with DMEM/F-12 (Gibco, USA), and then non-solid fraction of the bone marrow was put into 15 mL centrifuge tubes and centrifuged for 5 min (1000 rpm). After washing with PBS, 4 mL DMEM/F-12 containing 10% FBS and 1% penicillin–streptomycin (Gibco, USA) were added to the cell pellet. Then the cells were resuspended and seeded onto petri dish cultured in 5% CO_2_ at 37 ℃. After 48–72 h, cell culture medium was replaced to remove non-adherent cells. When the cells reached 80–90% confluence, they were split, and subcultured at a density of roughly 2 × 10^6^ cells per culture dish. The morphology of BMSCs was observed with an IX73 inverted microscope (Olympus, Japan). All experiments performed in the present study were approved by the Animal Care and Ethics Committee of Huazhong University of Science and Technology.

### Ultrasound irradiation

BMSCs were exposed to ultrasound (US) by positioning the probe of a 1 MHz medical ultrasound transducer (Olympus, Japan) under the bottom of the culture dish (Additional file 1: Fig. S1). Parameters of US were as follows: intensity of 300 mW/cm^2^, duty cycle of 20%, acoustic frequency of 1 MHz, and repetition frequency of 100 Hz. We selected the acoustic frequency of 1 MHz for the future application to deep tissues, and duty cycle 20% to avoid excess heat generation. Different intensities (30, 100, 300 mW/cm^2^) of ultrasonic stimulation were applied on the cells. 300 mW/cm^2^, the maximum intensity to be set in our machine, was chosen, as it was believed that stronger cavitation could facilitate extracellular vesicle release [34], and we have verified that such intensity did not affect cell viability. Control samples were handled identically to US-treated samples but without irradiation. 24 h after US stimulation, the cells and conditioned medium were collected for subsequent analyses.

### Isolation and characterization of BMSCs-derived extracellular vesicles

EVs-depleted FBS (HYcezmbio, China) was used in cell culture for EVs isolation. Briefly, the conditioned medium of BMSCs was centrifuged at 300*g* for 10 min and then at 3000*g* for 10 min at 4 ℃. The supernatants were centrifuged at 13,000*g* at 4 ℃ for 30 min, and then at 120,000*g* at 4 ℃ for 70 min. The resulting precipitation was washed and resuspended in PBS, followed by centrifugation at 120,000*g* at 4 ℃ for 70 min. The EVs pellet was then resuspended in PBS. The concentration and size distribution of BMSCs-derived EVs were measured by a Nanosight NS300 (Malvern, UK). Transmission electron microscopy (TEM) was used to identify EVs morphology. The characteristic proteins of EVs including CD9, CD63, CD81 and TSG101 (Abcam, UK) were analyzed by western blotting.

### Animal model

BALB/c (H-2d) was used as donors, and C57BL/6 (H-2b) as recipients. Male 8 ~ 12-week-old mice (20–25 g) were purchased from and housed in Tongji Medical School Animal Care Facility. All animal experiments were performed with the approval of the Institutional Animal Care and Use Committee of the Tongji Medical College, Huazhong University of Science and Technology (Wuhan, China) under the Guide for the Care and Use of Laboratory Animals of the National Institutes of Health. A full-thickness trunk skin graft was harvested from a BALB/c donor mouse. 1 cm^2^ of left upper back skin was removed from the recipient C57BL/6 mouse. For both C-EVs and LIPUS-EVs groups, 50 μg of EVs solution was dripped on the wound of the recipient C57BL/6 mouse. Harvested BALB/c donor skin was trimmed, placed onto the EVs solution and sutured. Transplanted skin was secured with a bandage for 7 days.

### Cell culture, LPS and extracellular vesicles treatment

RAW264.7 cells (Chinese Academy of Sciences Cell Bank of Type Culture Collection (CBTCCCAS, Shanghai, China), TCM13) were cultured to 60–70%. Then they were randomly divided into four groups: (1) the control group, (2) LPS group, (3) LPS + C-EVs group, and (4) LPS + LIPUS-EVs group. The cells in the control group were pretreated with PBS and cells in another three groups were treated with LPS (100 ng/mL) for 3 h [35]. Then the cells in LPS + C-EVs group and LPS + LIPUS-EVs group were treated with 15 μg/mL C-EVs and LIPUS-EVs, respectively, for 20 h [36].

### Cell viability assay

BMSCs were seeded into a 12-well plate with a density of 3 × 10^5^ cells/well and incubated overnight. Then the cells were treated with different intensities of LIPUS (30, 100, 300 mW/cm^2^). After 6 h, 24 h and 48 h incubation, cell viability was assessed using 50 μL of Cell Counting Kit-8 (CCK-8, MedChemExpress, USA) according to the manufacturer’s instructions. RAW264.7 cells were seeded at a density of 5000 cells/well in a 96 well-plate and incubated at 37 °C overnight. Then, cells were treated with various final concentrations of LPS (25, 50, 75, 100, 125, 150, 200, 250 ng/mL). After treatment with LPS for 24 and 48 h, 10 μL of CCK-8 was added to each well and incubated for 2 h. The absorbance of the resulting color, formazan, at 450 nm was assayed using a multimode microplate reader (TECAN, Switzerland). Cell viability of RAW264.7 cells treated with different concentrations of C-EVs and LIPUS-EVs (10, 15, 20, 25 μg/mL) was assessed by the CCK-8 assay in a similar manner.

### Zeta potential measurements

The zeta potential (ZP) analysis was performed using ZetaView nanoinstrument (Particle Metrix, Germany). The ZP of EVs was measured three times at 25 °C under the following settings: sensitivity of 85, a shutter value of 70, and a frame rate of 30 frames per second.

### Flow cytometry analysis

The surface markers expression of BMSCs isolated from mice was analyzed by flow cytometry (BD FACSCanto™, USA), including CD44, CD29, Sca-1, CD45, CD34 and CD11b (BD Biosciences, 1:100, USA) [37].

### Histologic analysis

Skin grafts were fixed in formalin and embedded to paraffin block. Samples were cut into 5-μm thick sections and stained with hematoxylin and eosin (H&E). Sections were stained with CD11b (Abcam, #ab133357; 1:2000, UK), CD68 (BOSTER biological technology, #BA3638; 1:300, China), CD3 (Abcam, #ab16669; 1:100, UK). Subsequently, sections were visualized under an IX73 inverted microscope (Olympus, Japan).

### Quantitative real-time PCR analysis (qRT-PCR)

RNA of samples was extracted with TRIzol reagent (Vazyme, China), and first strand cDNA was synthesized using 1 μg of RNA and HiScript® III RT SuperMix for qPCR (Vazyme, China). RT-PCR was performed with SYBR Green PCR reagents (Vazyme, China) on a detection system (Bio-rad, USA). RNA levels were normalized to the level of GAPDH (for mRNA expression) and U6 (for miRNA expression) and calculated as delta-delta threshold cycle (ΔΔCT). Primers used for RT-PCR are listed below: GAPDH-For: TCGCTCCTGGAAGATGGTGAT, GAPDH-Rev: CAGTGGCAAAGTGGAGATTGTTG, NF-κB-For: ATGGCAGACGATGATCCCTAC, NF-κB-Rev: TGTTGACAGTGGTATTTCTGGTG, IL-6-For: ACAACCACGGCCTTCCCTACT, IL-6-Rev: CTCATTTCCACGATTTCCCAGA, IL-10-For: CTTACTGACTGGCATGAGGATCA, IL-10-Rev: GCAGCTCTAGGAGCATGTGG. The matured mouse miR-328-5p and miR-487b-3p levels were determined using corresponding Bulge-Loop™ miRNA qRT-PCR primer sets customized and purchased from RIBOBIO, China.

### Western blotting

Total protein was harvested using a RIPA Kit (Beyotime, China) with the addition of 1 mM protease inhibitor PMSF at 4 °C. The presence of EVs-specific markers was evaluated in the EVs samples by western blotting. Antibodies used in the study were as follows: CD9 (1:200, Duolaimi biotechnology, Wuhan, China), CD63 (1:200, Duolaimi biotechnology, Wuhan, China), CD81 (1:1000, Proteintech, USA), TSG101 (1:200, Duolaimi biotechnology, Wuhan, China), and Calnexin (1:5000, Proteintech, USA), Western blotting was performed with primary antibodies including anti-Alix (1:1000, Abcam, UK), anti-HGS (1:1000, Abcam, UK), anti-YBX-1 (1:1000, Abcam, UK), anti-LRP5 (1:1000, Abcam, UK), anti-MYC (1:1000, Abcam, UK), anti-NF-κB (1:1000, Abcam, UK), anti-TNF-α (1:1000, Abcam, UK) and GAPDH (diluted 1:5000) (Abcam, UK). Quantification of Western blotting data was performed using ImageJ (NIH, USA).

### Enzyme-linked immunosorbent assay (ELISA)

The levels of IL-10 and NF-κB in the skin tissues and RAW264.7 cells were evaluated using commercial enzyme-linked immunosorbent assay kits (HYcezmbio, China). All assays were repeated at least three times to confirm the reliability of the study.

### mRNA and miRNA analysis

The mRNA and miRNA analysis were conducted by Haplox (Jiang Xi, China). Total RNA was isolated from the untreated BMSCs, LIPUS-treated BMSCs, C-EVs and LIPUS-EVs using TRIzol reagent (Vazyme, China). The concentration, quality and integrity of the total RNA were determined with a Nano Drop 2000 spectrophotometer (ThermoFisher Scientific, USA) and an Agilent 2100 Bioanalyser (Agilent Technologies, USA). The total RNA that had a standard of concentration ≥ 200 ng/mL, mass ≥ 10 mg and RNA integrity number (RIN) ≥ 8.0 was subjected to RNA-Seq. The clean reads were obtained through quality control and then blasted against the Rfam database to annotate the miscellaneous RNAs [38]. After filtering snRNA, scRNA, rRNA, and other non-coding RNA, the remaining sRNAs were aligned to the miRBase version 21.0 to identify the known and novel miRNAs. Then, the differential miRNA expression profile was calculated by DESeq2 [39] and edgeR [40]. MiRNAs with |log2fold change|≥ 2 and *p*-value < 0.05 were identified as having statistical differences. Supervised clustering was used to generate a volcano plot and identify differentially expressed genes and novel isoforms. Gene Ontology (GO) enrichment analysis for biological processes was used to investigate specific GO terms. Kyoto Encyclopedia of Genes and Genomes (KEGG) pathway enrichment analysis was conducted to explore the most significantly enriched pathways for differentially expressed genes (DEGs) and differentially expressed miRNAs (DEMiRNAs). The procedure of RNA-seq was performed on Illumina Hiseq2500 platform according to the manufacturer's instructions Majorbio Biotech Co., Ltd. (Shanghai, China).

### Statistical analysis

All experiments were repeated at least three times to confirm the reliability of the study. Statistical analysis was performed by Student’s *t*-test (*α* = 0.05) or one-way analysis of variance (ANOVA) using GraphPad Prism version 8.0 (GraphPad Software, USA). All data were presented as the mean value ± standard deviation (SD). *p* < 0.05 were considered to be statistically significant.

## Results

### Characterization of BMSCs and BMSCs-derived extracellular vesicles

**Fig. 1:**
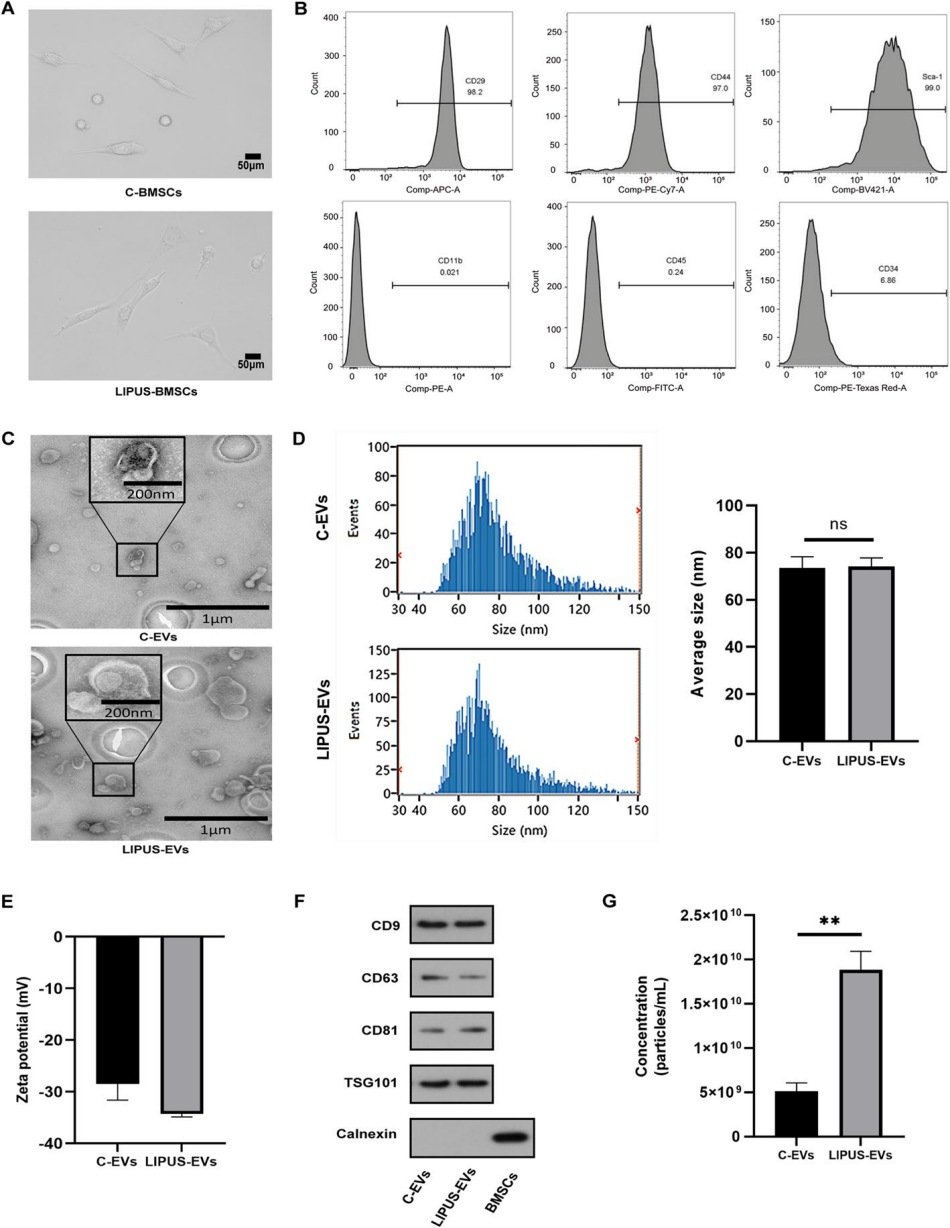
Characterization of BMSCs and BMSCs-derived EVs. **A** BMSCs before and after LIPUS irradiation both displayed a representative spindle-like morphology. Scale bar: 50 μm. **B** Flow cytometry analysis of the cell surface markers on BMSCs. Cells were cultured in P5 or P6 and the results showed that BMSCs were positive for CD29, CD44 and Sca-1, but negative for CD11b, CD45 and CD34. **C** Morphology of C-EVs and LIPUS-EVs observed by TEM. Scale bar, 1 μm; inset scale bar, 200 nm. **D** Average size of BMSCs-derived EVs measured by NanoSight NTA. *n* = 5 per group. **E** Zeta potential of C-EVs and LIPUS-EVs detected using ZetaView. *n* = 3 per group. **F** The surface markers of BMSCs-derived EVs (CD9, CD63, CD81, TSG101 and Calnexin) were detected by western blotting. **G** Overall concentration of EVs by Nanosight NTA profiling revealing a significant increase in EVs following LIPUS exposure**.** **p < 0.01 vs. Control. *n* = 5 per group. Statistical significance assessed by unpaired two-tailed *t*-test. EVs, extracellular vesicles

To study the effects of LIPUS stimulation on EVs production, BMSCs were harvested from the mouse bone marrow and cultured for in vitro and in vivo experiments and treated with LIPUS as described in Methods. The cells displayed a spindle-like shape which were observed under an optical microscope (Fig. 1A). Flow cytometry analysis suggested that the isolated BMSCs expressed mesenchymal positive markers CD29, CD44 and Sca-1, and presented negative expression of CD11b, CD45 and CD34 (Fig. 1B). The results from TEM indicated that the majority of these EVs secreted by LIPUS-BMSCs exhibited typical cup-shaped bilayer membrane structure which were identical to those produced by untreated BMSCs (Fig. 1C). The size of the isolated EVs was measured from Nanosight assay, showing a relatively uniform size distribution of particles with a mean diameter of 74.21 nm for LIPUS-EVs and 73.47 nm for C-EVs (Fig. 1D). The results showed that C-EVs had an average surface charge of − 28.48 ± 3.136 mV and LIPUS-EVs had an average surface charge of − 34.30 ± 0.5728 mV (Fig. 1E). There was no significant difference in ZP between C-EVs and LIPUS-EVs. Furthermore, these EVs were positive for EVs’ characteristic surface markers, including CD9, CD63, CD81 and TSG101, and negative for a cytosolic marker, Calnexin, detected by western blotting (Fig. 1F). By NTA analysis, it was further determined that exposure of BMSCs to LIPUS elicited a statistically significant increase of 3.66 times in LIPUS-EVs release compared to untreated controls (Fig. 1G).

### LIPUS promotes the proliferation, extracellular vesicles secretion and anti-inflammatory effects of BMSCs

**Fig. 2:**
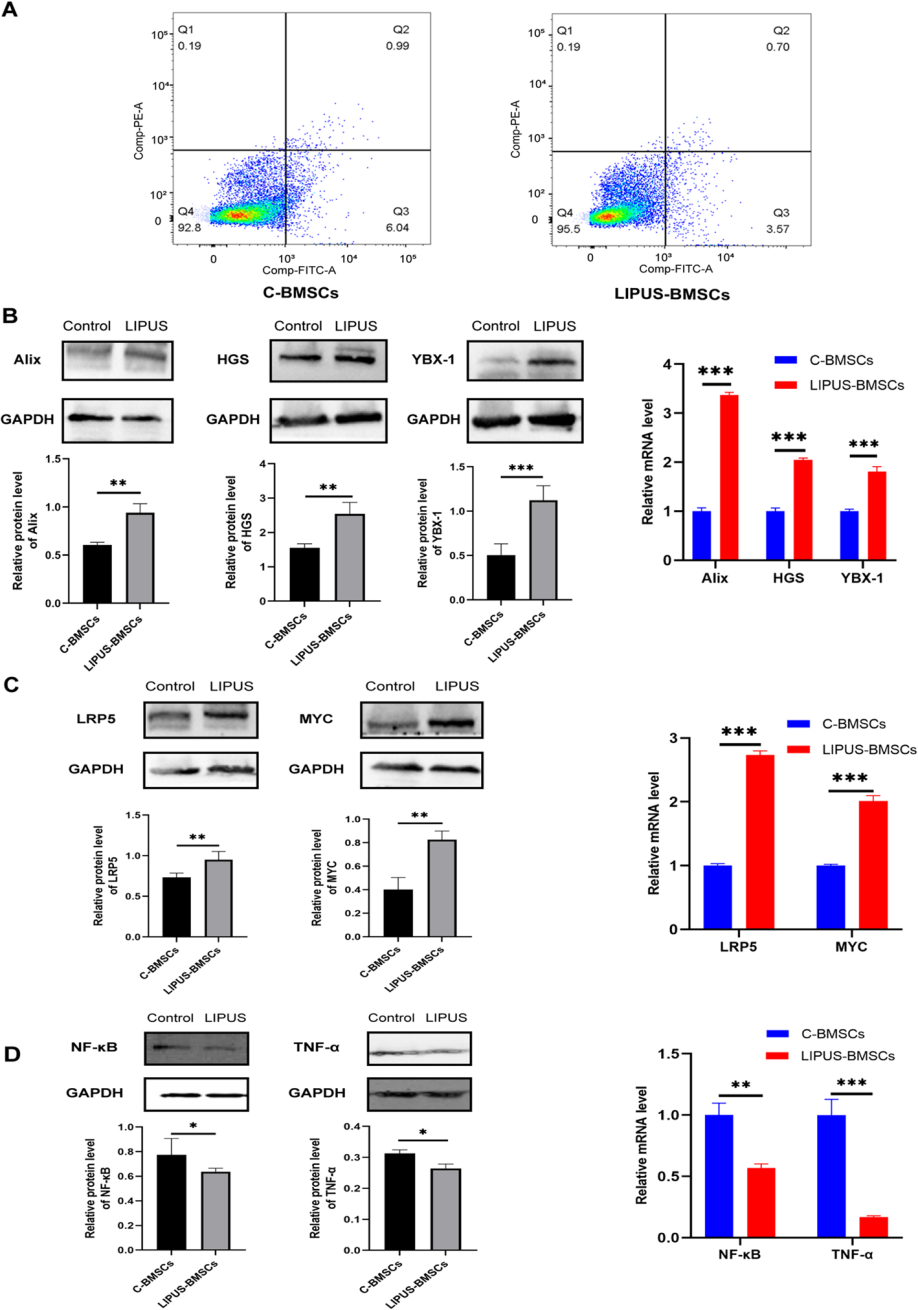
LIPUS promoted the proliferation, EVs secretion and anti-inflammatory effects of BMSCs. **A** Flow cytometry analysis of cell apoptosis for C-BMSCs and LIPUS-BMSCs. **B** Protein and mRNA expression of Alix, HGS and YBX-1 measured by western blotting and qPCR. **C** Protein and mRNA expression of LRP5 and MYC measured by western blotting and qPCR. **D** Protein and mRNA level of IL-8, NF-κB and TNF-α detected by western blot and qPCR, respectively. Error bars represent mean ± SD (*n* = 3 per group). ns *p* > 0.05, *p < 0.05, **p < 0.01, ***p < 0.001. Statistical significance assessed by unpaired two-tailed *t*-test. EVs, extracellular vesicles

The effect of LIPUS stimulation on BMSCs were measured, especially in EVs secretion, proliferation and anti-inflammatory functions. Flow cytometry analysis showed that LIPUS did not promote or inhibit apoptosis of BMSCs (Fig. 2A). Additionally, LIPUS treatment obviously up-regulated the protein and mRNA expression of Alix, HGS and YBX-1 which were genes involved in the biogenesis and cargo sorting of EVs (Fig. 2B). CCK-8 assays revealed that LIPUS with an intensity of 300 mW/cm^2^ promoted cell proliferation at 6, 24 and 48 h after LIPUS stimulation compared with control group (Additional file 2: Fig. S2). LIPUS stimulation also significantly increased the protein and mRNA expression of cell cycle associated genes, LRP5 and MYC, indicating that LIPUS could facilitate the proliferation of BMSCs (Fig. 2C). Moreover, we explored whether LIPUS could promote the anti-inflammatory function of BMSCs. Western blotting results showed that BMSCs stimulated by LIPUS had much lower protein expression of proinflammatory factors, NF-κB and TNF-α. In addition, qPCR further verified the above changes in the mRNA level of NF-κB and TNF-α (Fig. 2D). In brief, the above results revealed that LIPUS-treated BMSCs were more prone to be anti-inflammatory than untreated BMSCs. LIPUS promoted the EVs secretion and proliferation of BMSCs and modulated BMSCs to be a more anti-inflammatory phenotype than untreated ones.

### LIPUS strengthened the anti-inflammatory effect of BMSC-derived extracellular vesicles

**Fig. 3:**
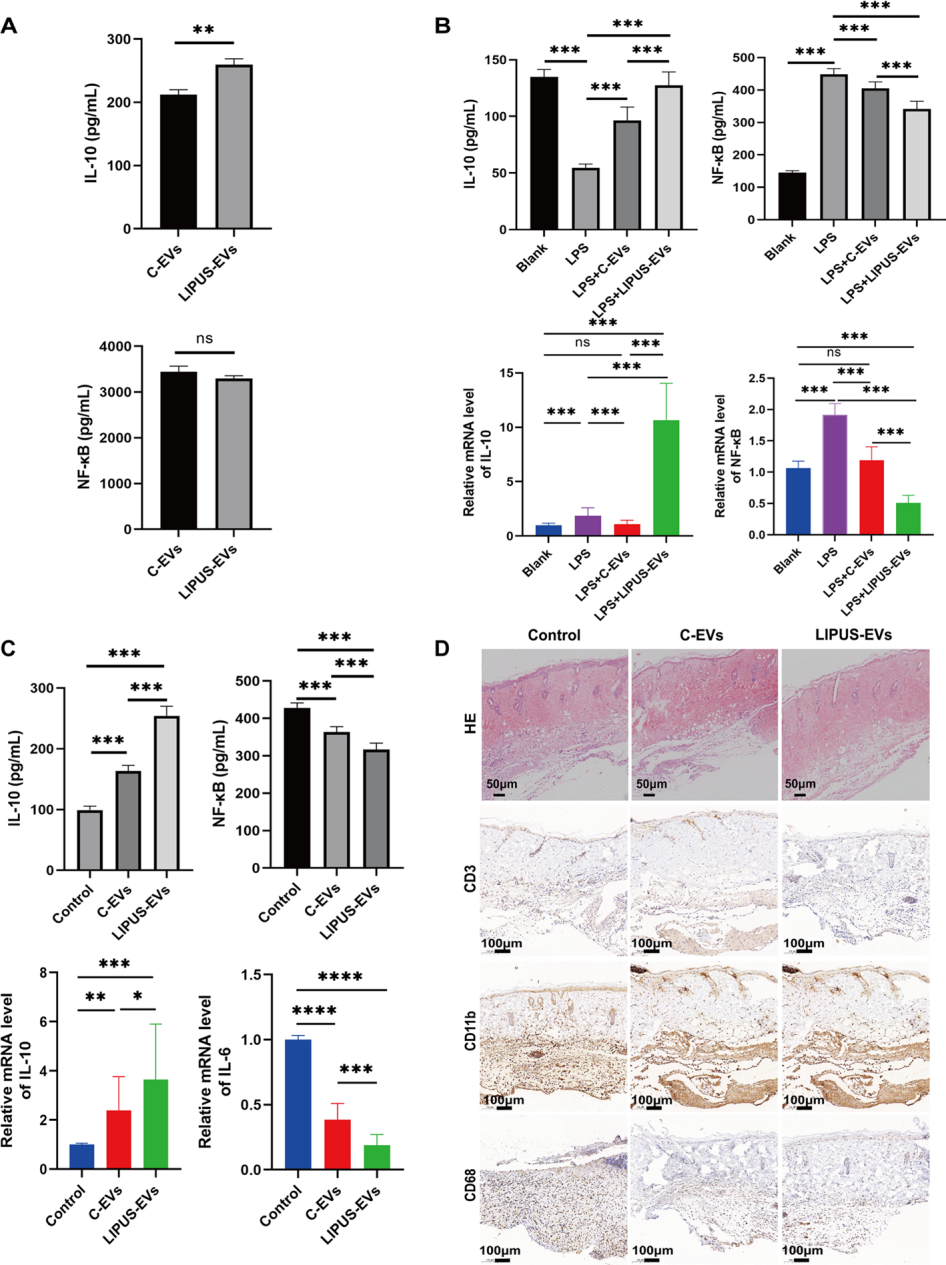
LIPUS strengthened the anti-inflammatory effect of BMSCs-derived EVs. **A** The protein expression levels of pro-inflammatory cytokines (IL-10 and NF-κB) in both C-EVs and LIPUS-EVs detected by ELISA assay (*n* = 6 per group). **B** The protein expression of IL-10 and NF-κB in RAW264.7 cells measured by ELISA. And the mRNA level of IL-10 and NF-κB in RAW264.7 cells by qRT-PCR. (*n* = 6 per group). **C** The protein expression of IL-10 and NF-κB in skin allografts measured by ELISA. The mRNA expression of IL-10 and IL-6 in skin allografts measured by qRT-PCR. (*n* = 4 per group). **D** H&E-stained sections of skin allograft. Scale bar = 50 μm. Immunohistochemistry staining of the skin allograft in control group contained massive infiltrates of CD3^+^, CD11b^+^ and CD68^+^ cells in comparison to the C-EVs and LIPUS-EVs group. (Representative images from 3 different mice per group). Scale bar = 100 μm. Error bars represent mean ± SD. ns *p* > 0.05, *p < 0.05, **p < 0.01, ***p < 0.001. Statistical significance assessed by unpaired two-tailed *t*-test. EVs, extracellular vesicles

To explore the influence of LIPUS stimulation on the anti-inflammatory effect by BMSCs-derived EVs, the relative expression of anti-inflammatory factor (IL-10) and pro-inflammatory factor NF-κB in C-EVs and LIPUS-EVs were determined using ELISA. As shown in Fig. 3A, the results from ELISA showed that LIPUS stimulation significantly increased the expression of anti-inflammatory factor (IL-10) in BMSCs derived EVs, albeit there was no significant difference in NF-κB. Moreover, in order to compare the anti-inflammatory function of C-EVs and LIPUS-EVs, two models of inflammation were used. The in vitro model, LPS treated mouse macrophage RAW264.7 cells, was a mimic for infectious inflammation. In order to ascertain whether LPS and EVs exert toxicity on RAW264.7 cells, the CCK-8 assay was performed. As shown in Additional file 3: Fig. S3 and Additional file 4: Fig. S4 the treatment with LPS (100 ng/mL) and EVs (15 μg/mL) for 24 and 48 h did not significantly harm the cell viability. And the in vivo model, mouse skin transplantation, included both infectious and sterile inflammation [41, 42]. The protein and mRNA expression of IL-10 were obviously down-regulated, while those of NF-κB were up-regulated in the LPS treated RAW 264.7 cells compared to the blank group (Fig. 3B). The protein and mRNA level of IL-10 were increased in the LPS + LIPUS-EVs group compared to LPS group (*p* < 0.001, with 5.69 folds mRNA expression and 2.32 folds protein expression in LIPUS-EVs vs. LPS), and the degree of such increase was higher than that of the LPS + C-EVs group (*p* < 0.001, with 1.76 folds protein expression in C-EVs vs. LPS). The protein and mRNA level of NF-κB both significantly decreased in the LPS + LIPUS-EVs group compared to LPS group (*p* < 0.001, with 0.266 folds mRNA expression and 0.762 folds protein expression in LIPUS-EVs vs. LPS), and the degree of such decrease was higher than that of the LPS + C-EVs group (*p* < 0.005, with 0.621 folds mRNA expression and 0.902 folds protein expression in C-EVs vs. LPS) (Fig. 3B).

To investigate the in vivo effect of LIPUS-EVs, a well-established mouse model of skin transplantation was selected. In this model, the protein and mRNA level of IL-10 were increased in the LIPUS-EVs group compared to control group (*p* < 0.001, with 3.65 folds mRNA expression and 2.57 folds protein expression in LIPUS-EVs vs. control), and the degree of such increase was higher than that of the C-EVs group (*p* < 0.01, with 2.39 folds mRNA expression and *p* < 0.001, with 1.65 folds protein expression in C-EVs vs. control). The protein level of NF-κB was decreased in the LIPUS-EVs group compared to control group (*p* < 0.001, with 0.74 folds protein expression in LIPUS-EVs vs. control), and the degree of such decrease was higher than that of the C-EVs group (*p* < 0.001, with 0.85 folds protein expression in C-EVs vs. control) (Fig. 3C). There was no significant difference in mRNA levels of NF-κB among the three groups (Additional file 5: Fig. S5). The mRNA level of IL-6 was decreased in the LIPUS-EVs group compared to control group (*p* < 0.001, with 0.19 folds mRNA expression in LIPUS-EVs vs. control), and the degree of such decrease was higher than that of the C-EVs group (*p* < 0.001, with 0.38 folds mRNA expression in C-EVs vs. control) (Fig. 3C). To further validate these findings, the presence of specific immune cells and skin-related markers was assessed by H&E staining and immunohistochemical staining. As the representative images shown in Fig. 3D, the margin between the skin of the recipient and the allograft in control group contained massive cellular infiltration, indicating active inflammation. In comparison, the cellular infiltration was less dense at the site of attachment between the skin allograft transplanted and the skin of the recipient in EVs treated groups. There were numerous CD3^+^ cells infiltration in the control group, suggesting massive T cells recruitment to the skin allograft. Compared to C group, less CD3^+^ cells were found in the C-EVs group, indicating the anti-inflammation effect of BMSCs-derived EVs. Since LIPUS-EVs group showed significantly decreased CD3^+^ cells even compared to C-EVs group, we speculated that LIPUS stimulation enhanced the anti-inflammatory effect of BMSCs-derived EVs. Furthermore, immunohistochemical staining results of CD11b^+^, CD68^+^ and CD169^+^ (Additional file 6: Fig. S6), three markers of macrophages, showed similar results as CD3^+^. There were more infiltrated macrophages infiltration in the skin allograft of control group, and less in C-EVs group, much less in LIPUS-EVs group (Fig. 3D). Based on these results, it was obvious that BMSCs-derived EVs ameliorated inflammation in both in vitro and in vivo models, and LIPUS stimulation further strengthened the anti-inflammatory effects of BMSCs-derived EVs.

### Analysis of differentially expressed genes between C-BMSCs and LIPUS-BMSCs with functional annotation and classification

**Fig. 4:**
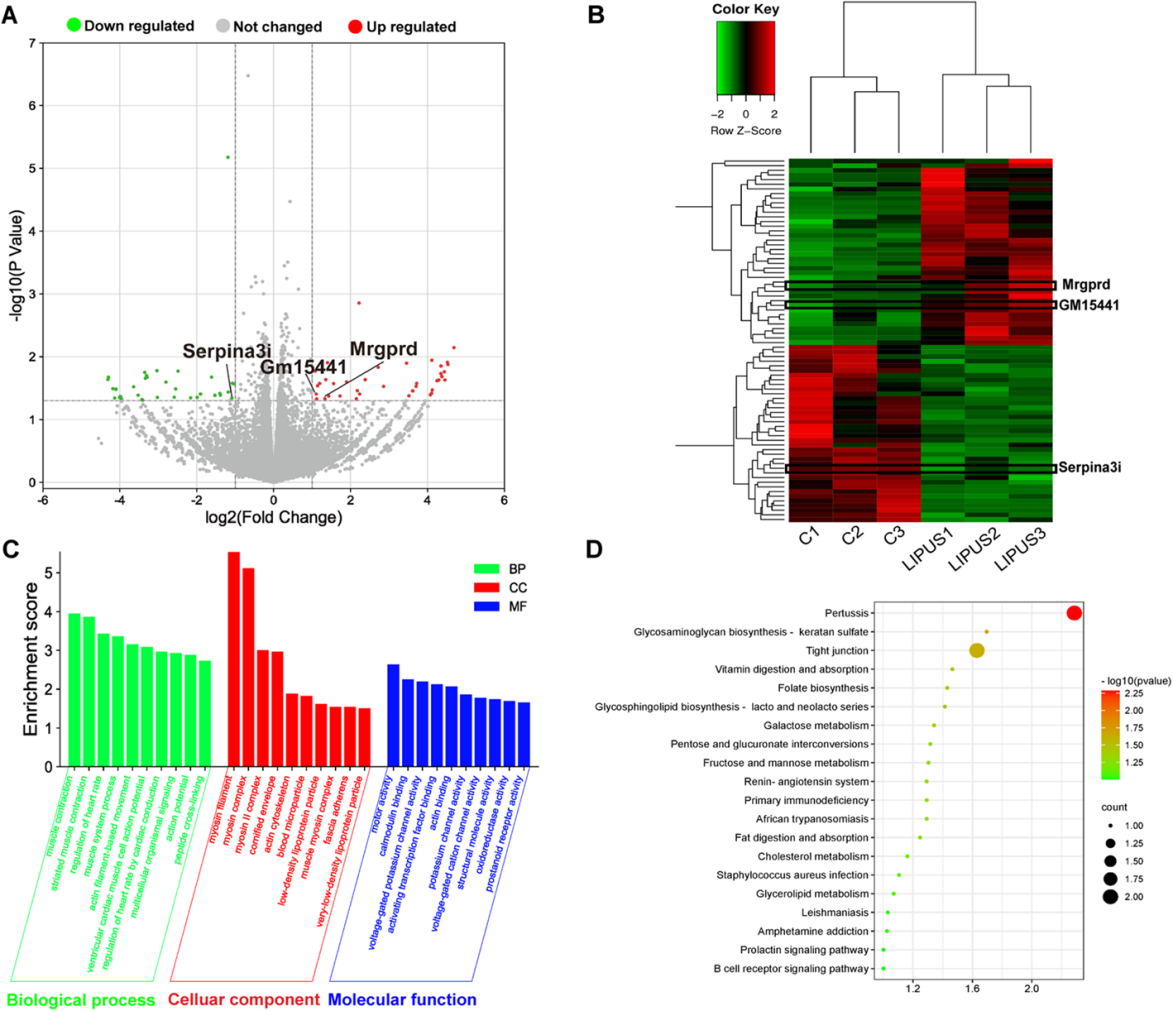
Analysis of differentially expressed genes between C-BMSCs and LIPUS-BMSCs with functional annotation and classification. **A** Volcano map of DEGs between C-BMSCs and LIPUS-BMSCs. The *x*-axis is the log2 scale of the fold change of gene expression in MSCs and induced ECs (log2(fold change)). Negative values indicate downregulation; positive values indicate upregulation. The *y*-axis is the minus log10 scale of the adjusted *p* values (− log10 (*p* value)), which indicate the significant level of expression difference. The red dots represent significantly upregulated genes with at least twofold change, while the green dots represent significantly downregulated genes with at least twofold change. **B** Heatmap of the DEGs between C-BMSCs and LIPUS-BMSCs. Red stripes in the Figure represent high expression genes, while green stripes represent low expression genes. **C** Significant enriched GO terms between C-BMSCs and LIPUS-BMSCs based on their functions. **D** The top 20 enriched KEGG pathway terms of DEGs

To reveal the molecular mechanism associated with LIPUS-stimulation, a DEG analysis was performed to identify gene expression changes between C-BMSCs and LIPUS-BMSCs. A total of 78 DEGs (The cutoff values are fold change > 2 and *p* < 0.05) were detected between the C-BMSCs and LIPUS-BMSCs, of which 40 genes were upregulated (higher expression in LIPUS-BMSCs) and 38 genes were downregulated (Fig. 4A, B). We identified two genes (Gm15441, Mrgprd) that were significantly upregulated and one (Serpina3i) downregulated (over 10 folds) in the LIPUS-BMSCs compared with C-BMSCs.

GO analyses were made on three different aspects namely biological process (BP), cellular component (CC), and molecular function (MF), reflecting the dynamic alteration processes during LIPUS stimulation (Fig. 4C). According to the functional enrichment results, the most enriched BP terms include muscle contraction, striated muscle contraction and regulation of heart rate. Myosin filament and myosin complex were the most enriched CC terms. The most enriched MF terms were motor activity, calmodulin binding and voltage-gated potassium channel activity. In addition, a KEGG pathway enrichment analysis was conducted to explore the most significantly enriched pathways for DEGs (Fig. 4D), and the results showed these target genes were mainly involved in the tight junction, primary immunodeficiency and B cell receptor signaling pathways. These results suggest that LIPUS-BMSCs enriched genes may play regulatory roles in these pathways.

### Identification of differentially expressed miRNAs between C-EVs and LIPUS-EVs and functional analysis of DEmiRNAs using GO and KEGG pathway analysis

**Fig. 5:**
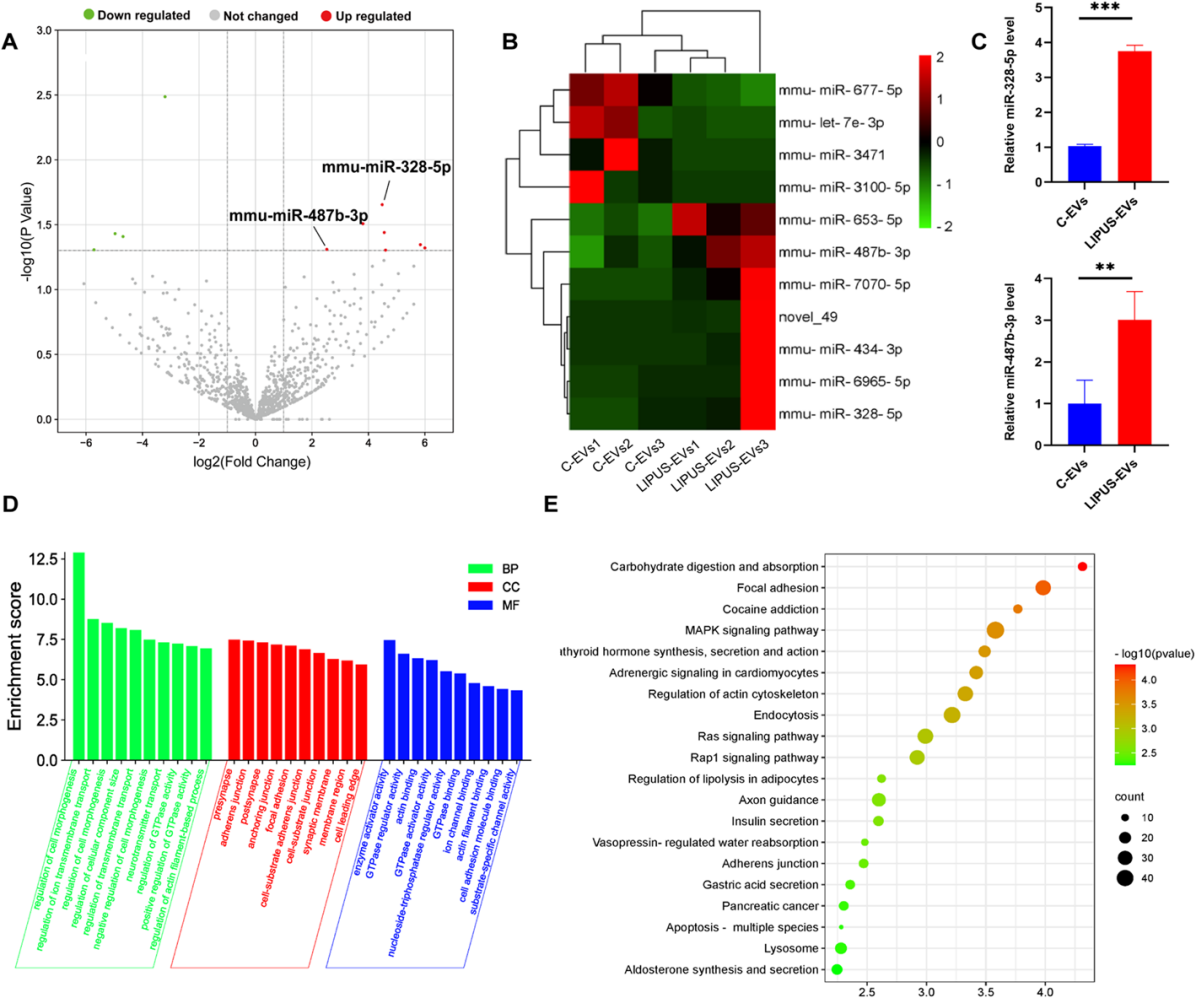
Identification of differentially expressed miRNAs between C-EVs and LIPUS-EVs and functional analysis of DEmiRNAs using GO and KEGG pathway analyses. **A** Volcano map of DEmiRNAs between C-EVs and LIPUS-EVs. The *x*-axis is the log2 scale of the fold change of gene expression in C-EVs and LIPUS-EVs (log_2_(fold change)). Negative values indicate downregulation; positive values indicate upregulation. The *y*-axis is the minus log10 scale of the adjusted *p* values (− log10 (*P* value)), which indicates the significant level of expression difference. The red dots represent significantly upregulated genes with at least twofold change, while the green dots represent significantly downregulated genes with at least twofold change. **B** Heatmap of the differentially expressed genes between C-EVs and LIPUS-EVs. Red stripes in the figure represent high expression genes, while green stripes represent low expression genes. **C** qRT-PCR analysis of miR-328-5p and miR-487b-3p. *n* = 3 per group. ***p < 0.001, **p < 0.01. **D** Significant enriched GO terms between C-EVs and LIPUS-EVs based on their functions. **E** Top 20 KEGG pathways enriched in target genes of DEmiRNAs. The *x*-axis indicates the ratio of the number of genes in the DEmiRNAs, and the *y*-axis indicates the pathways. *EVs* extracellular vesicles

In order to capture global gene expression changes associated with LIPUS-stimulation in BMSCs-derived EVs, RNA-seq data were generated from the untreated and LIPUS-stimulated BMSCs-derived EVs, with three biological replicates, respectively. Using a fold change cutoff of 2 and a *p*-value cutoff of 0.05, we identified a total of 11 differentially expressed miRNAs (DEmiRNAs) (Fig. 5A). Unsupervised hierarchical clustering analysis revealed that 7 DEmiRNAs were up-regulated and 4 DEmiRNAs were down-regulated in the LIPUS-EVs (Fig. 5B). Among them, miR-328-5p and miR-487b-3p were found to be commonly enriched in LIPUS-EVs but not C-EVs. To confirm the reliability of the expression profiles generated using the RNA-Seq data, qRT-PCR was applied to examine the expression levels of the 2 up-regulated candidate miRNAs (miR-328-5p and miR-487b-3p) in LIPUS-EVs compared with C-EVs. Consistent with the RNA-seq data, miR-328-5p miRNA level, measured by RT-qPCR, was significantly increased (*p* < 0.001) in LIPUS-EVs (Fig. 5C). Similarly, the level of miR-487b-3p miRNA was significantly increased (*p* < 0.01) in LIPUS-EVs (Fig. 5C). As expected, the qRT-PCR results basically matched the RNA-seq results and validated the expression pattern of two DEmiRNAs in the RNA-Seq data.

To understand the regulatory functions of miRNA specifically expressed in LIPUS-EVs, we performed GO and KEGG pathway analyses and found several miRNAs and their target functional genes which were differentially expressed from LIPUS-EVs and C-EVs (Additional file 7: Table S1 and Additional file 8: Table S2). Enriched GO terms were grouped in the three categories: biological process (BP), cellular component (CC), and molecular function (MF), respectively (Fig. 5D). In the BP category, the most enriched terms included regulation of cell morphogenesis, ion transmembrane transport and cellular component size. The most enriched CC term were presynapse and adherens junction. The most enriched MF terms were enzyme activator activity, GTPase regulator activity and actin binding. Additionally, a KEGG analysis was performed, and the results showed that the target genes of these miRNAs were mainly involved in the MAPK, Ras and Rap1 signaling pathways (Fig. 5E).

## Discussion

BMSCs-derived EVs have shown potent anti-inflammatory function in various pathological conditions, such as osteoarthritis and neurodegenerative diseases [1, 3]. Since the number of EVs naturally secreted by cells is finite and they usually bear specific repertoires of bioactive molecules to perform manifold cell–cell communication, but not one particular therapeutic function as expected, their practical application is still limited [17, 22, 23]. Strategies are needed to increase the production of EVs and enhance their therapeutic function. Recent studies have suggested that LIPUS is a promising non-invasive method to regulate EVs production and promote their anti-inflammatory effect [27, 43]. However, the effect of LIPUS stimulation of BMSCs on EVs derived from the cells remains unclear. In this study, we investigated whether LIPUS stimulation on BMSCs could increase the secretion of EVs and enhance their anti-inflammatory effects. The EVs secreted from LIPUS-treated BMSCs exhibited classic features of EVs. LIPUS treatment not only led to a 3.66-fold increase in the EVs release from BMSCs but also remarkably changed the amounts of certain components packaged in EVs, namely, increased miR-328-5p and miR-487b-3p. Moreover, both in vitro and in vivo experimental results suggested that LIPUS-EVs possessed stronger anti-inflammatory function than C-EVs.

As a tool for regulating BMSCs’ EVs production, LIPUS has a number of advantages: non-invasiveness, convenience, and low cost. The non-invasive feature was confirmed by our results in Fig. 1A–C, which showed that LIPUS treatment did not affect the morphology of BMSCs nor did it induce apoptosis of the cells. Moreover, our results in Fig. 2A-B suggested that LIPUS stimulation had several positive effects on BMSCs. It significantly increased expression of genes related to EVs biogenesis (Alix and HGS) [44, 45], EVs cargo sorting (YBX-1) [46], and cell cycle (LRP5 and MYC) [47–49]. Additionally, LIPUS could significantly downregulated pro-inflammatory factors, i.e., NF-κB and TNF-α, as shown in Fig. 2C. These changes of BMSCs after LIPUS treatment may provide the basis for changes in EVs derived from LIPUS treated BMSCs.

It is worth noting that LIPUS irradiation has led to a 3.66-fold increase of EVs secretion from BMSCs as shown in Fig. 1G. In order to reveal the mechanism underlying such increase, an RNA-seq was conducted using BMSCs with/without LIPUS stimulation. DEGs between control and LIPUS groups were identified and functional enrichment analyses were carried out. The most enriched BP terms included muscle contraction, striated muscle contraction and regulation of heart rate. Myosin filament and myosin complex were the most enriched CC terms. The most enriched MF terms were motor activity, calmodulin binding and voltage-gated potassium channel activity. With relatively low intensity, LIPUS is non-invasive and it mainly performs its bioeffects through its non-thermal effects, predominantly cavitation, acoustic streaming, and acoustic radiation force [50]. It has been reported that cells are capable of sensing these external mechanical stimuli and transduce them into biochemical signals, triggering downstream pathways [51]. Cytoskeleton plays an important role in many cellular activities, including intracellular vesicle transportation, mitogenesis, and EVs biogenesis [52]. The GO functional enrichment analysis result showed that DEGs were mainly involved in myosin filament, myosin complex, motor activity, and calmodulin binding. These cellular components and molecular functions were all involved in cytoskeleton activity. Therefore, these results indicated that LIPUS stimulation may enhance the cytoskeleton activity of BMSCs, thus promoting the secretion activity, including EVs production, of the cells.

Under normal physiological conditions, MSCs reside in a perivascular location [31]. Once a local injury happens, they become activated and secrete bioactive molecules to regulate the local immune response [53]. These findings suggest that MSCs perform their therapeutic/medicinal effects mainly through paracrine function [54]. As the major component of MSCs’ secretome, EVs transfer bioactive molecules, such as mRNA, miRNA, DNA and proteins, between cells [55, 56]. MicroRNAs (miRNAs), 19–24 nucleotide small non-coding RNAs, can regulate gene expression post-transcriptionally by mediating hydrolysis and translation inhibition of targeted mRNAs [57]. And it is estimated that miRNAs regulate more than 60% of all mRNAs [58]. It is not surprising that EVs-contained miRNAs are significant regulators of immune cells’ function and play key roles in the regulation of inflammatory responses [59]. Therefore, in order to investigate how LIPUS irradiation modulate the therapeutic effect of MSCs’ EVs, we identified the differential expression of miRNA in the EVs derived from MSCs with/without LIPUS stimulation using small RNA-seq.

RNA-seq results showed that 7 miRNAs were upregulated in LIPUS-EVs, and 4 miRNAs were downregulated in LIPUS-EVs (Fig. 5A, B). Moreover, enrichment analyses were carried out for target genes of DEmiRNAs. The most enriched terms of GO pathway analysis were involved in intracellular activity, such as GTPase regulator activity and cellular component size, and intercellular connection, such as adherens junction. These results suggested that the DEmiRNAs of LIPUS-EVs participate in both intra- and inter-cellular activities. In addition, the target genes of DEmiRNAs mainly participate in the MAPK, Ras and Rap1 signaling pathways as shown in KEGG pathway analysis (Fig. 5E). Since Rap1 and Ras signaling pathways are both involved in MAPK pathway, the latter one should play a central role [60]. Considering the unambiguous link between MAPK pathway and inflammatory pathways [61], we speculated that the enhanced anti-inflammatory effects of LIPUS-EVs were mainly attributed to the suppression of genes related to pro-inflammatory MAPK pathway by those DEmiRNAs upregulated in LIPUS-EVs, such as miR-328-5p [62] and miR-487b-3p [63].

In order to compare the anti-inflammatory function of C-EVs and LIPUS-EVs, two models of inflammation were used. The in vitro model, LPS treated mouse macrophage RAW264.7 cells, was a mimic for infectious inflammation. And the in vivo model, mouse skin transplantation, included both infectious and sterile inflammation. Due to the sophisticated techniques and sterile surgical environment, sterile inflammation may play a key role in host defense after skin transplantation. Although infection is incited by invading microbes and sterile inflammation is stimulated by sterile stimuli such as mechanical trauma and ischemia, these divergent triggers ultimately result in the same downstream cellular manifestations of inflammation [64]. The NF-κB pathway has a central role in orchestrating the expression of multitudinous genes that control both innate and adaptive immune responses [65]. Since NF-κB is a pivotal regulator of pro-inflammatory gene expression, the protein expression level of NF-κB was used as an indicator of inflammation in this study. On the other hand, since IL-10 suppresses the production of proinflammatory cytokines [66], the expression level of IL-10 was used as an indicator of anti-inflammation.

Our results showed that BMSCs-derived EVs markedly attenuated inflammation in two models, manifested as decreased expression of proinflammatory factors and increased expression of anti-inflammatory factors, as well as less immune cells infiltration in mouse recipient with skin transplantation (Fig. 3). Moreover, LIPUS stimulation could further enhance such anti-inflammation effects of BMSCs-derived EVs. Based on analysis of the RNA-seq data, two upregulated genes (Gm15441, Mrgprd) and one downregulated gene (Serpina3i) in LIPUS-BMSCs may be the reason such enhancement, as their up-/down-regulation can suppress inflammation through different mechanisms [67–69] (Additional file 9: Fig. S7). More importantly, two upregulated miRNAs (miR-328-5p and miR-487b-3p) in LIPUS-EVs can target MAPK signaling pathway, which is also an important player in inflammation [70]. Although further investigation is needed to verify these speculated mechanisms, these results provide a theoretical foundation for applying LIPUS as a non-invasive strategy to manipulate BMSCs derived EVs aiming to increase their production as well as enhance their therapeutical function, such as the anti-inflammatory effect. Thus, the clinical translation of such cell-free therapy can be fostered.

## Conclusion

We conducted a preliminary study to investigate the effect of LIPUS on the yield and anti-inflammatory function of BMSCs-derived EVs and further explore the potential mechanism underneath. The results suggested that LIPUS stimulation could lead to a 3.66-fold increase increase in the EVs release from BMSCs. Moreover, both in vitro and in vivo experimental results suggested that LIPUS-EVs possessed stronger anti-inflammatory function than C-EVs. RNA-seq analysis revealed that miR-328-5p and miR-487b-3p were significantly up-regulated in LIPUS-EVs compare with C-EVs. The suppression of MAPK signaling pathway by these two up-regulated miRNAs could be the potential mechanism of strengthened anti-inflammatory effects of LIPUS-EVs. Therefore, LIPUS could be a promising non-invasive strategy to increase the production of EVs from BMSCs and enhance their anti-inflammatory effects.

### Supplementary Information

**Additional file 1: Fig. S1.** Schematic representation of LIPUS irradiation system. The platform includes a function generator (Tektronix, USA), a power amplifier (T&C Power Conversion, Inc., USA), a 10-cm culture dish, a 6-cm high agarose phantom, and an ultrasonic transducer fixed by a tube clamp.

**Additional file 2: Fig. S2.** Cell proliferation and viability of BMSCs exposed to LIPUS at different intensities and different time points determined by CCK-8 assay. A. Cell proliferation of BMSCs exposed to LIPUS at different intensities (30, 100, 300 mW/cm^2^) and different time points (6, 24, 48 h) determined by CCK-8 assay. B. Cell viability of BMSCs exposed to LIPUS at different intensities and different time points.

**Additional file 3: Fig. S3.** Viability of RAW264.7 cells exposed to LPS at different concentrations. Cell viability of RAW264.7 cells exposed to LPS at different concentrations (25, 50, 75, 100, 125, 150, 200, 250 ng/mL) at different time points (24, 48 h) determined by CCK-8 assay.

**Additional file 4: Fig. S4.** Viability of RAW264.7 cells exposed to C-EVs and LIPUS-EVs at different concentrations. Cell viability of RAW264.7 cells exposed to C-EVs (A) and LIPUS-EVs (B) at different intensities (10, 15, 20, 25 μg/mL) and different time points (24, 48 h) determined by CCK-8 assay.

**Additional file 5: Fig. S5.** The mRNA expression of NF-κB in skin allografts measured by qRT-PCR. The mRNA expression of NF-κB in skin allografts measured by qRT-PCR showed no significant difference among different groups. Error bars represent mean ± SD. Statistical significance assessed by unpaired two-tailed *t*-test. (*n* = 4 per group).

**Additional file 6: Fig. S6. **CD169 + immunohistochemical staining of skin allografts. Control group contained massive infiltration of CD169 + cells in comparison to the C-EVs and LIPUS-EVs group. Scale bar = 50 μm. (Representative images from 3 different mice per group).

**Additional file 7: Table S1.** MiRDB target prediction data_mmu-miR-328-5p.

**Additional file 8: Table S2.** MiRDB target prediction data_mmu-miR-487b-3p.

**Additional file 9: Fig. S7.** Schematic diagram illustrating the mechanism of LIPUS stimulation enhancing the anti-inflammatory effects of BMSCs-derived extracellular vesicles via the MAPK pathway in LPS-induced RAW264.7 cells. LPS triggered the inflammatory environment by enhancing the MAPK signaling pathway, while LIPUS upregulated genes (Gm15441, Mrgprd) and miRNAs (miR-328-5p, miR-487b-3p), downregulated one gene (Serpina3i) and hence reduce inflammation. (This figure was created by Figdraw).

## Data Availability

All data generated or analyzed during this study are included in this published article and its supplementary information files. The raw data are available from the corresponding author upon reasonable request.

## Abbreviations

LIPUS: Low-intensity pulsed ultrasound
BMSCs: Bone marrow mesenchymal stem cells
EVs: Extracellular vesicles
NTA: Nano tracking analysis
PBS: Phosphate buffered saline
IL: Interleukin
TNF: Tumor necrosis factor
GVHD: Graft versus-host disease
BMDCs: Bone marrow dendritic cells
LPS: Lipopolysaccharide
NF-κB: Nuclear factor- kappa B
FBS: Fetal bovine serum
US: Ultrasound
TEM: Transmission electron microscopy
BCA: Bicinchoninic acid
TBS: Tris buffered saline
HRP: Horseradish peroxidase
ECL: Enhanced chemiluminescence
RIN: RNA integrity number
GO: Gene ontology
KEGG: Kyoto encyclopedia of genes and genomes
DEGs: Differentially expressed genes
DEMiRNAs: Differentially expressed miRNAs
BP: Biological process
CC: Cellular component
MF: Molecular function
qRT-PCR: Quantitative real-time PCR analysis
ELISA: Enzyme-linked immunosorbent assay
