# Correction: Low-intensity pulsed ultrasound (LIPUS) enhances the anti-inflammatory effects of bone marrow mesenchymal stem cells (BMSCs)-derived extracellular vesicles

**DOI:** 10.1186/s11658-024-00620-7

**Published:** 2024-07-02

**Authors:** Xueke Li, Yi Zhong, Wuqi Zhou, Yishu Song, Wenqu Li, Qiaofeng Jin, Tang Gao, Li Zhang, Mingxing Xie

**Affiliations:** 1 Department of Ultrasound Medicine, Union Hospital, Tongji Medical College, Huazhong University of Science and Technology, Wuhan, 430022 China; 2 Clinical Research Center for Medical Imaging in Hubei Province, Wuhan, 430022 China; 3 Hubei Province Key Laboratory of Molecular Imaging, Wuhan, 430022 China

**Correction: Cellular & Molecular Biology Letters (2023) 28:9** 10.1186/s11658-023-00422-3

Following publication of the original article [1], the authors identified an error in Fig. 3D. The pictures used in the C-EVs and LIPUS-EVs groups of CD11b^+^ in Fig. 3D were inadvertently placed by mistake. We have double checked the original data and found that the inadvertent errors occurred during picture compilation, and this correction does not affect the scientific conclusions of the article.

The correct Fig. 3 is given in this correction.

**Fig. 3:**
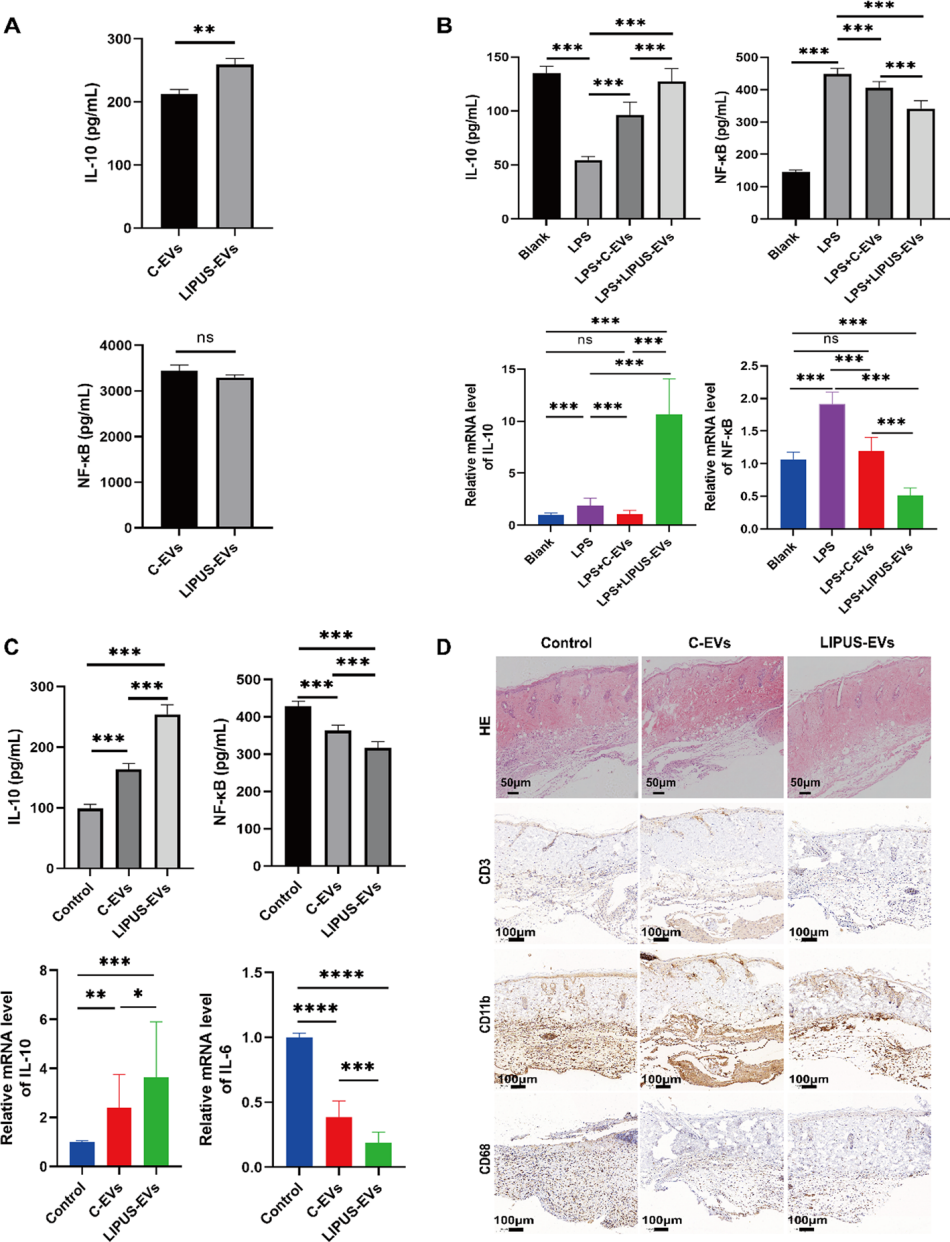
LIPUS strengthened the anti-inflammatory effect of BMSCs-derived EVs. **A** The protein expression levels of pro-inflammatory cytokines (IL-10 and NF-κB) in both C-EVs and LIPUS-EVs detected by ELISA assay (n = 6 per group). **B** The protein expression of IL-10 and NF-κB in RAW264.7 cells measured by ELISA. And the mRNA level of IL-10 and NF-κB in RAW264.7 cells by qRT-PCR. n = 6 per group. **C** The protein expression of IL-10 and NF-κB in skin allografts measured by ELISA. The mRNA expression of IL-10 and IL-6 in skin allografts measured by qRT-PCR. n = 4 per group. **D** H&E-stained sections of skin allograft. Scale bar = 50 μm. Immunohistochemistry staining of the skin allograft in control group contained massive infiltrates of CD3^+^, CD11b^+^ and CD68^+^ cells in comparison to the C-EVs and LIPUS-EVs group. Representative images from 3 different mice per group. Scale bar = 100 μm. Error bars represent mean ± SD. ns p > 0.05, *p < 0.05, **p < 0.01, ***p < 0.001. Statistical significance assessed by unpaired two-tailed t-test. *EVs* extracellular vesicles
