# Supplementary figures and images for: Low-intensity pulsed ultrasound (LIPUS) enhances the anti-inflammatory effects of bone marrow mesenchymal stem cells (BMSCs)-derived extracellular vesicles
Source: Cell Mol Biol Lett. 2023 Jan 30;28:9. doi: 10.1186/s11658-023-00422-3 (PMC9885645; doi:10.1186/s11658-023-00422-3)

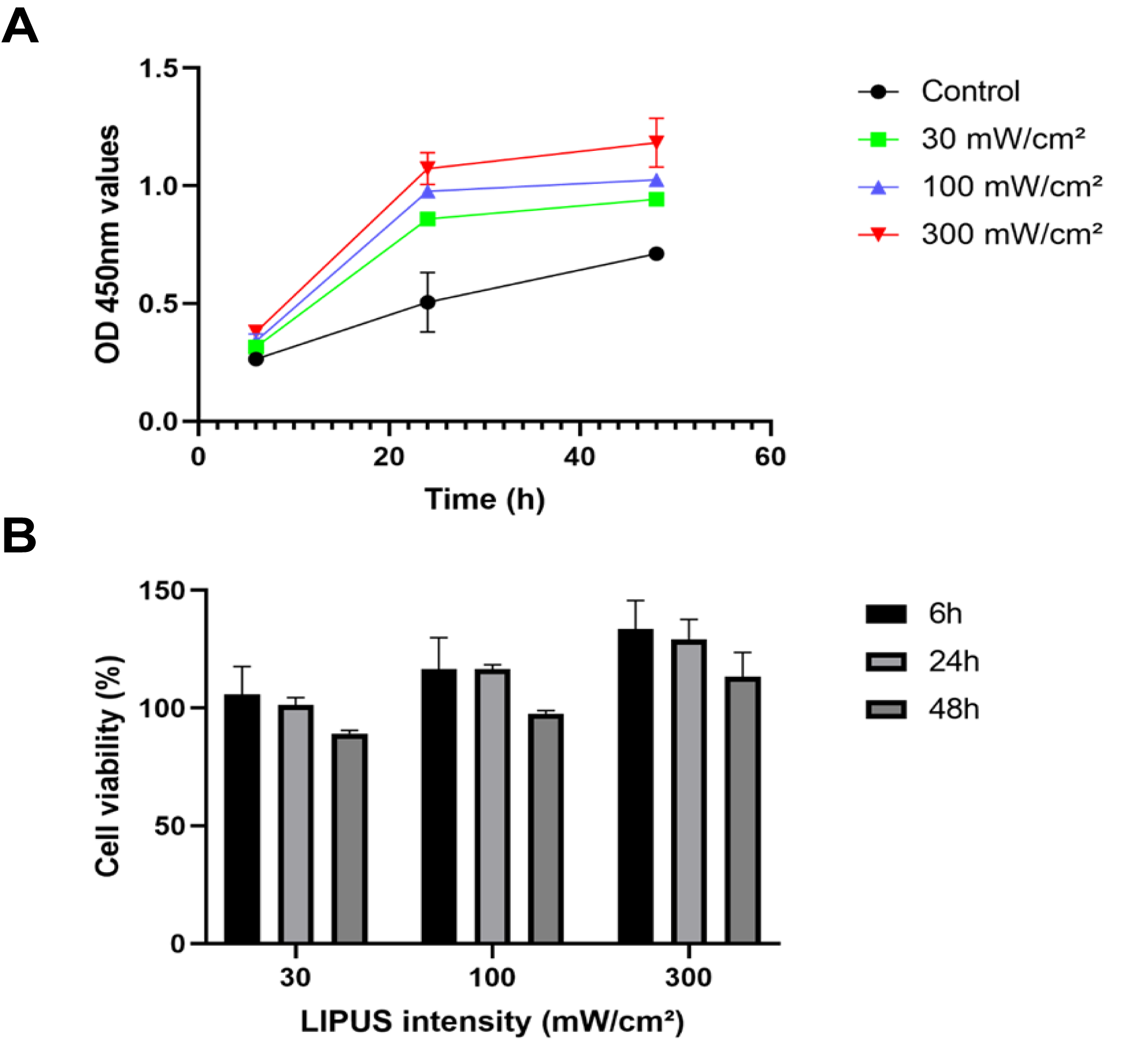

Supplement: Supplementary file 2 — Additional file 2: Fig. S2. Cell proliferation and viability of BMSCs exposed to LIPUS at different intensities and different time points determined by CCK-8 assay. A. Cell proliferation of BMSCs exposed to LIPUS at different intensities (30, 100, 300 mW/cm2) and different time points (6, 24, 48 h) determined by CCK-8 assay. B. Cell viability of BMSCs exposed to LIPUS at different intensities and different time points. [file 11658_2023_422_MOESM2_ESM.tif]

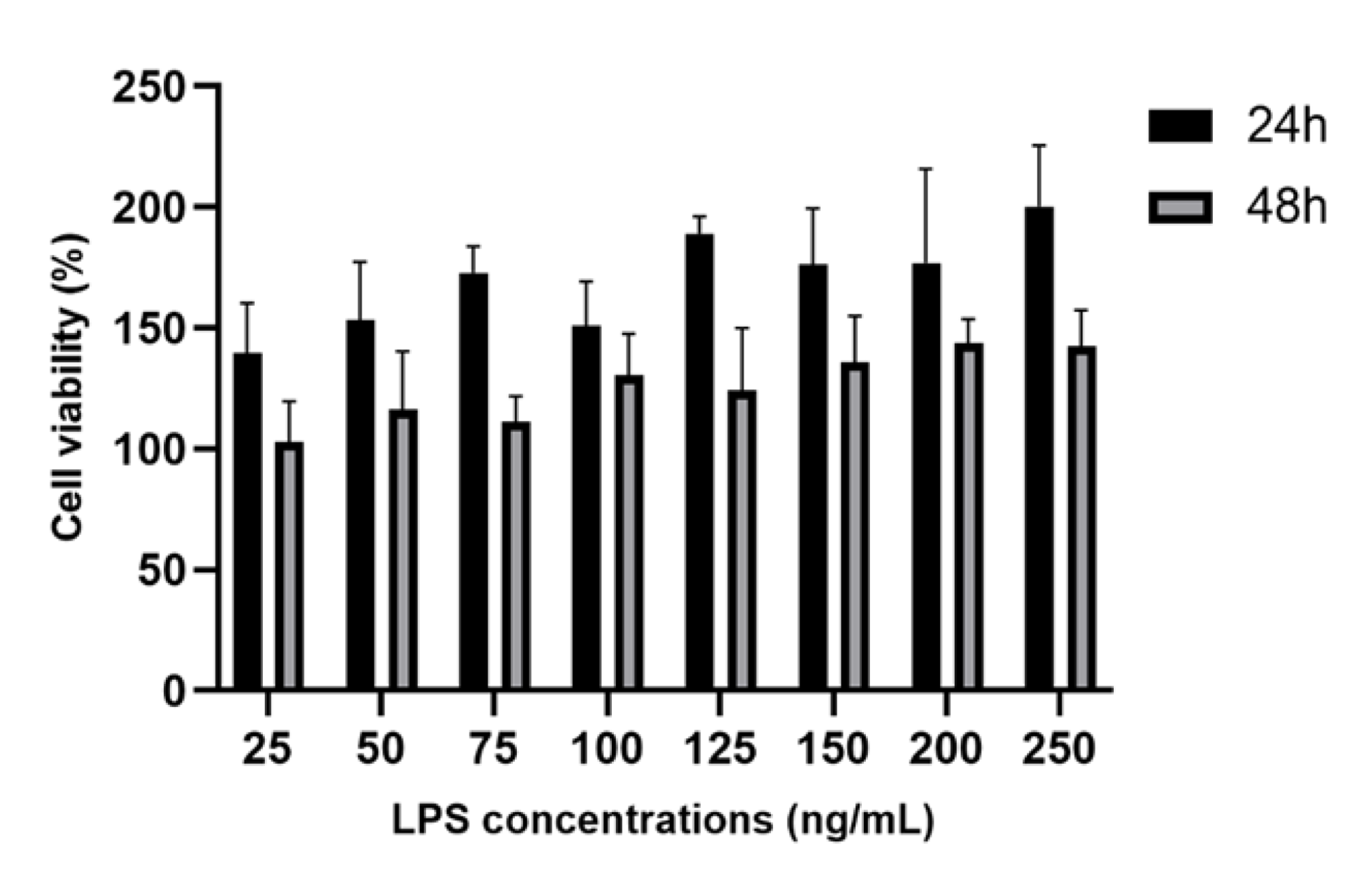

Supplement: Supplementary file 3 — Additional file 3: Fig. S3. Viability of RAW264.7 cells exposed to LPS at different concentrations. Cell viability of RAW264.7 cells exposed to LPS at different concentrations (25, 50, 75, 100, 125, 150, 200, 250 ng/mL) at different time points (24, 48 h) determined by CCK-8 assay. [file 11658_2023_422_MOESM3_ESM.tif]

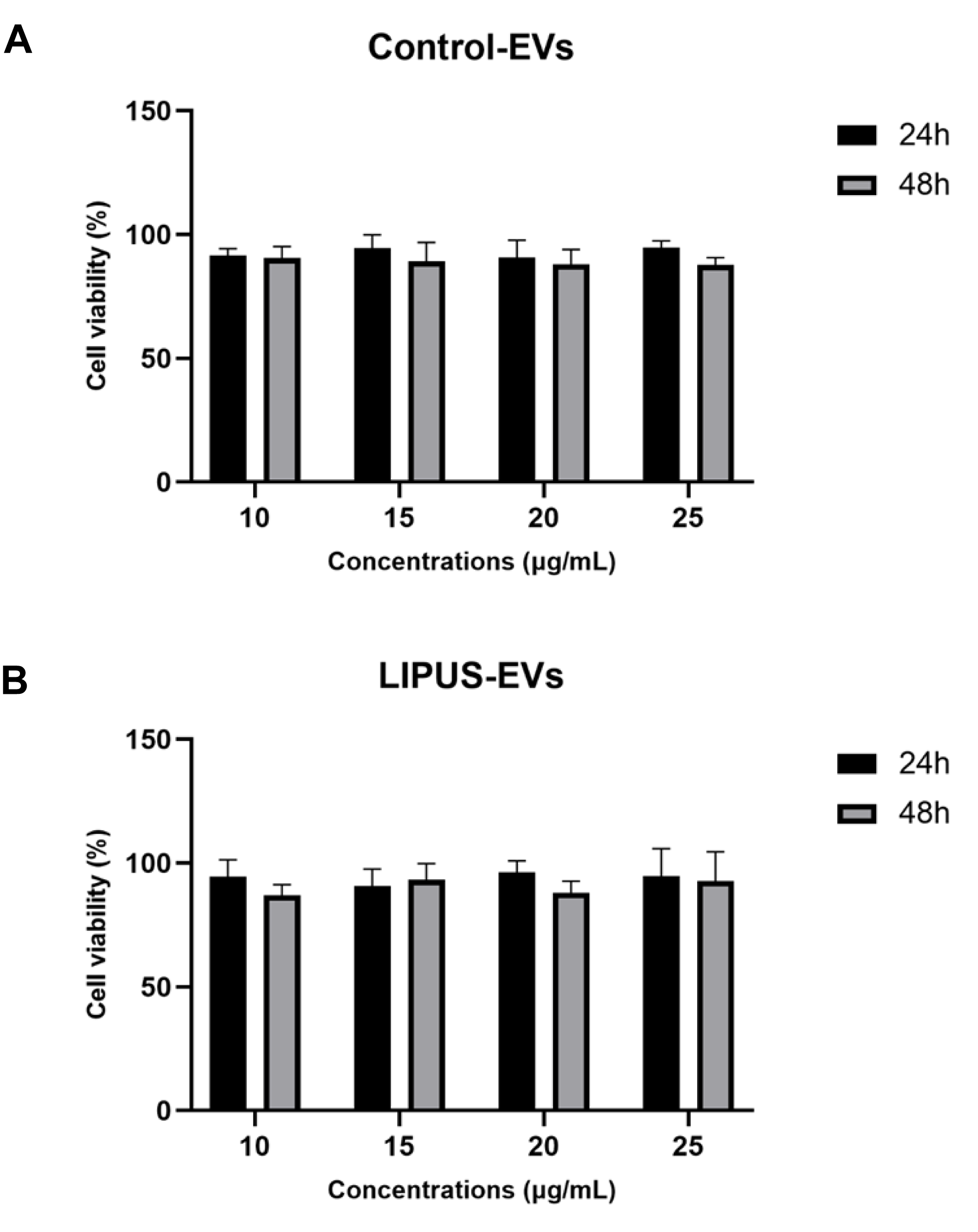

Supplement: Supplementary file 4 — Additional file 4: Fig. S4. Viability of RAW264.7 cells exposed to C-EVs and LIPUS-EVs at different concentrations. Cell viability of RAW264.7 cells exposed to C-EVs (A) and LIPUS-EVs (B) at different intensities (10, 15, 20, 25 μg/mL) and different time points (24, 48 h) determined by CCK-8 assay. [file 11658_2023_422_MOESM4_ESM.tif]

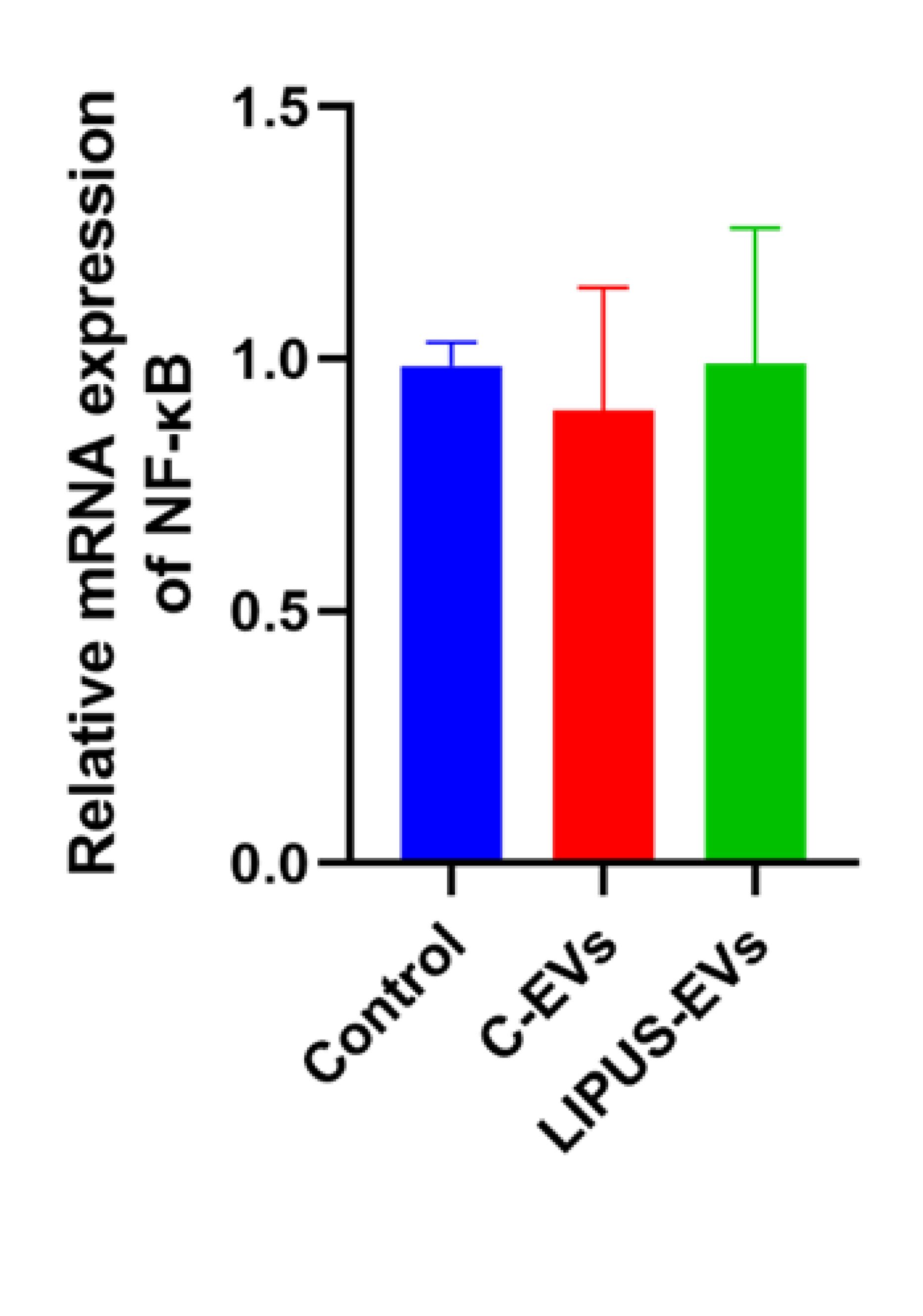

Supplement: Supplementary file 5 — Additional file 5: Fig. S5. The mRNA expression of NF-κB in skin allografts measured by qRT-PCR. The mRNA expression of NF-κB in skin allografts measured by qRT-PCR showed no significant difference among different groups. Error bars represent mean ± SD. Statistical significance assessed by unpaired two-tailed t-test. (n = 4 per group). [file 11658_2023_422_MOESM5_ESM.tif]

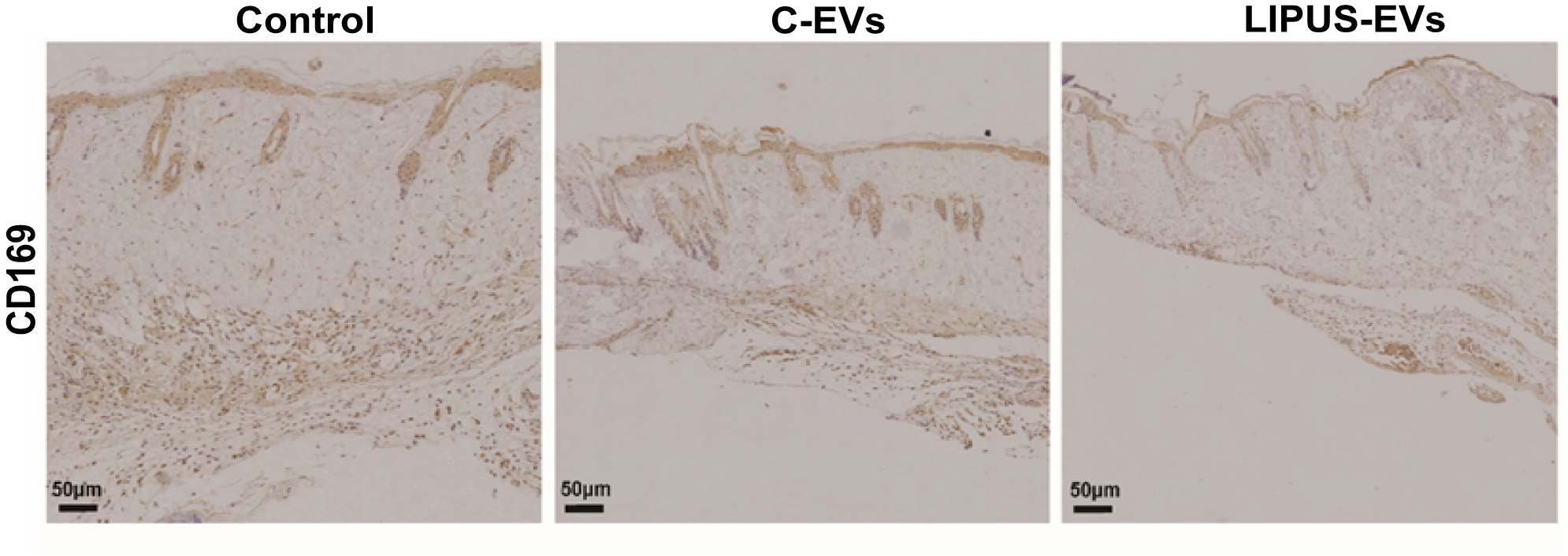

Supplement: Supplementary file 6 — Additional file 6: Fig. S6. CD169 + immunohistochemical staining of skin allografts. Control group contained massive infiltration of CD169 + cells in comparison to the C-EVs and LIPUS-EVs group. Scale bar = 50 μm. (Representative images from 3 different mice per group). [file 11658_2023_422_MOESM6_ESM.tif]

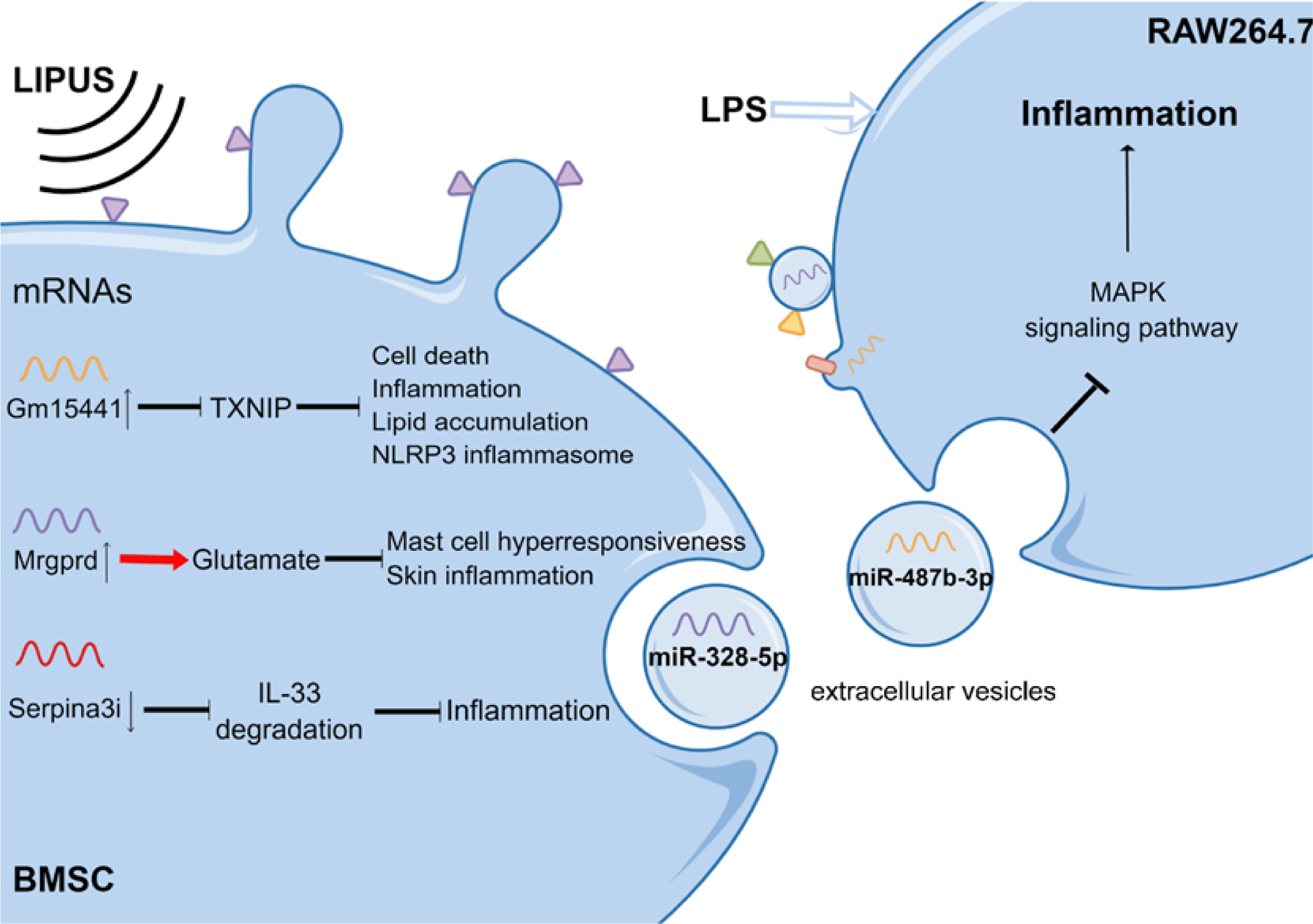

Supplement: Supplementary file 9 — Additional file 9: Fig. S7. Schematic diagram illustrating the mechanism of LIPUS stimulation enhancing the anti-inflammatory effects of BMSCs-derived extracellular vesicles via the MAPK pathway in LPS-induced RAW264.7 cells. LPS triggered the inflammatory environment by enhancing the MAPK signaling pathway, while LIPUS upregulated genes (Gm15441, Mrgprd) and miRNAs (miR-328-5p, miR-487b-3p), downregulated one gene (Serpina3i) and hence reduce inflammation. (This figure was created by Figdraw). [file 11658_2023_422_MOESM9_ESM.tif]
