# Supplementary material for: Low-intensity pulsed ultrasound (LIPUS) enhances the anti-inflammatory effects of bone marrow mesenchymal stem cells (BMSCs)-derived extracellular vesicles
Source: Cell Mol Biol Lett. 2023 Jan 30;28:9. doi: 10.1186/s11658-023-00422-3 (PMC9885645; doi:10.1186/s11658-023-00422-3)
Supplement: Supplementary file 7 — Additional file 7: Table S1. MiRDB target prediction data_mmu-miR-328-5p. [file 11658_2023_422_MOESM7_ESM.docx]

**Table S1.** **MiRDB target prediction data_mmu-miR-328-5p.**

| **Target Rank** | **Target Score** | **Gene ID** | **Gene Symbol** | **Transcript Accession** | **Gene Description** |
| --- | --- | --- | --- | --- | --- |
| 1 | 98 | 209773 | Dennd2a | NM_172477 | DENN/MADD domain containing 2A |
| 2 | 96 | 70727 | Rasgef1a | NM_027526 | RasGEF domain family, member 1A |
| 3 | 95 | 56212 | Rhog | NM_019566 | ras homolog family member G |
| 4 | 94 | 12890 | Cplx2 | NM_009946 | complexin 2 |
| 5 | 92 | 27224 | Eloa | NM_013736 | elongin A |
| 6 | 92 | 12444 | Ccnd2 | NM_009829 | cyclin D2 |
| 7 | 92 | 56542 | Ick | NM_019987 | intestinal cell kinase |
| 8 | 92 | 268445 | Ankrd13b | NM_172945 | ankyrin repeat domain 13b |
| 9 | 92 | 18591 | Pdgfb | NM_011057 | platelet derived growth factor, B polypeptide |
| 10 | 91 | 16498 | Kcnab2 | NM_001252654 | potassium voltage-gated channel, shaker-related subfamily, beta member 2 |
| 11 | 91 | 11905 | Serpinc1 | NM_080844 | serine (or cysteine) peptidase inhibitor, clade C (antithrombin), member 1 |
| 12 | 90 | 14123 | Fbrs | NM_010183 | fibrosin |
| 13 | 90 | 209683 | Ttc28 | NM_001267622 | tetratricopeptide repeat domain 28 |
| 14 | 90 | 101809 | Spred3 | NM_182927 | sprouty-related, EVH1 domain containing 3 |
| 15 | 90 | 215751 | Ginm1 | NM_145418 | glycoprotein integral membrane 1 |
| 16 | 89 | 235044 | Plppr2 | NM_001290299 | phospholipid phosphatase related 2 |
| 17 | 88 | 21788 | Tfpi | NM_001177319 | tissue factor pathway inhibitor |
| 18 | 88 | 170733 | Klra17 | NM_133203 | killer cell lectin-like receptor, subfamily A, member 17 |
| 19 | 88 | 77630 | Prdm8 | NM_029947 | PR domain containing 8 |
| 20 | 88 | 229542 | Gatad2b | NM_139304 | GATA zinc finger domain containing 2B |
| 21 | 88 | 14842 | Gsx1 | NM_008178 | GS homeobox 1 |
| 22 | 88 | 70527 | Stambp | NM_024239 | STAM binding protein |
| 23 | 88 | 22318 | Vamp2 | NM_009497 | vesicle-associated membrane protein 2 |
| 24 | 87 | 78928 | Pigt | NM_133779 | phosphatidylinositol glycan anchor biosynthesis, class T |
| 25 | 87 | 11419 | Asic1 | NM_001289791 | acid-sensing (proton-gated) ion channel 1 |
| 26 | 87 | 16402 | Itga5 | NM_010577 | integrin alpha 5 (fibronectin receptor alpha) |
| 27 | 86 | 327766 | Tmem26 | NM_177794 | transmembrane protein 26 |
| 28 | 86 | 170574 | Sp7 | NM_130458 | Sp7 transcription factor 7 |
| 29 | 85 | 214897 | Csnk1g1 | NM_173185 | casein kinase 1, gamma 1 |
| 30 | 85 | 19357 | Rad21 | NM_009009 | RAD21 cohesin complex component |
| 31 | 84 | 78339 | Ttyh3 | NM_001308040 | tweety family member 3 |
| 32 | 84 | 67864 | Yipf4 | NM_026417 | Yip1 domain family, member 4 |
| 33 | 84 | 17347 | Mknk2 | NM_021462 | MAP kinase-interacting serine/threonine kinase 2 |
| 34 | 83 | 270120 | Fat3 | NM_001080814 | FAT atypical cadherin 3 |
| 35 | 83 | 380702 | Shisa6 | NM_001034874 | shisa family member 6 |
| 36 | 83 | 67379 | Dedd2 | NM_207677 | death effector domain-containing DNA binding protein 2 |
| 37 | 82 | 13641 | Efnb1 | NM_010110 | ephrin B1 |
| 38 | 82 | 211739 | Vstm2a | NM_001290539 | V-set and transmembrane domain containing 2A |
| 39 | 82 | 331537 | Pih1h3b | NM_177921 | PIH1 domain containing 3B |
| 40 | 81 | 81601 | Kat5 | NM_001199248 | K(lysine) acetyltransferase 5 |
| 41 | 81 | 72630 | Hspa12b | NM_028306 | heat shock protein 12B |
| 42 | 81 | 71972 | Dnmbp | NM_001306088 | dynamin binding protein |
| 43 | 81 | 66213 | Med7 | NM_001104556 | mediator complex subunit 7 |
| 44 | 81 | 52064 | Coq5 | NM_026504 | coenzyme Q5 methyltransferase |
| 45 | 81 | 382056 | Crtc1 | NM_001004062 | CREB regulated transcription coactivator 1 |
| 46 | 80 | 381813 | Prmt8 | NM_201371 | protein arginine N-methyltransferase 8 |
| 47 | 80 | 72508 | Rps6kb1 | NM_028259 | ribosomal protein S6 kinase, polypeptide 1 |
| 48 | 80 | 109050 | Inka2 | NM_175398 | inka box actin regulator 2 |
| 49 | 80 | 57743 | Sec61a2 | NM_021305 | Sec61, alpha subunit 2 (S. cerevisiae) |
