# Supplementary material for: Low-intensity pulsed ultrasound (LIPUS) enhances the anti-inflammatory effects of bone marrow mesenchymal stem cells (BMSCs)-derived extracellular vesicles
Source: Cell Mol Biol Lett. 2023 Jan 30;28:9. doi: 10.1186/s11658-023-00422-3 (PMC9885645; doi:10.1186/s11658-023-00422-3)
Supplement: Supplementary file 8 — Additional file 8: Table S2. MiRDB target prediction data_mmu-miR-487b-3p. [file 11658_2023_422_MOESM8_ESM.docx]

**Table S2.** **MiRDB target prediction data_mmu-miR-487b-3p**

| **Target Rank** | **Target Score** | **Gene ID** | **Gene Symbol** | **Transcript Accession** | **Gene Description** | |
| --- | --- | --- | --- | --- | --- | --- |
| 1 | 95 | 56876 | Nsmf | NM_020276 | NMDA receptor synaptonuclear signaling and neuronal migration factor |  |
| 2 | 93 | 70799 | Cep192 | NM_027556 | centrosomal protein 192 |  |
| 3 | 93 | 18750 | Prkca | NM_011101 | protein kinase C, alpha |  |
| 4 | 87 | 170772 | Glcci1 | NM_001286728 | glucocorticoid induced transcript 1 |  |
| 5 | 82 | 67122 | Nrarp | NM_025980 | Notch-regulated ankyrin repeat protein |  |
| 6 | 80 | 77125 | Il33 | NM_001164724 | interleukin 33 |  |
| 7 | 78 | 241656 | Pak7 | NM_172858 | p21 (RAC1) activated kinase 7 |  |
| 8 | 74 | 272589 | Tbcel | NM_173038 | tubulin folding cofactor E-like |  |
| 9 | 74 | 72475 | Ssbp3 | NM_198438 | single-stranded DNA binding protein 3 |  |
| 10 | 72 | 18741 | Pitx2 | NM_011098 | paired-like homeodomain transcription factor 2 |  |
| 11 | 70 | 51786 | Cpsf2 | NM_016856 | cleavage and polyadenylation specific factor 2 |  |
| 12 | 69 | 56324 | Stam2 | NM_019667 | signal transducing adaptor molecule (SH3 domain and ITAM motif) 2 |  |
| 13 | 67 | 228788 | Ccm2l | NM_145536 | cerebral cavernous malformation 2-like |  |
| 14 | 66 | 22771 | Zic1 | NM_009573 | zinc finger protein of the cerebellum 1 |  |
| 15 | 65 | 108907 | Nusap1 | NM_001042652 | nucleolar and spindle associated protein 1 |  |
| 16 | 63 | 20539 | Slc7a5 | NM_011404 | solute carrier family 7 (cationic amino acid transporter, y+ system), member 5 |  |
| 17 | 62 | 14525 | Gcsam | NM_001159297 | germinal center associated, signaling and motility |  |
